# Dispersion of Mechanical Properties of High-Strength Glass Fibre Composites in Hygrothermal Environment

**DOI:** 10.3390/polym14173514

**Published:** 2022-08-27

**Authors:** Xiang Wang, Bo Wang, Yu Zhang, Yongyong Suo, Purong Jia, Feng Huang

**Affiliations:** 1 School of Mechanics, Civil Engineering and Architecture, Northwestern Polytechnical University, Xi’an 710129, China; 2 Beijing Key Laboratory of Aeronautical Materials Testing and Evalution, AECC Beijing Institute of Aeronautical Materials, Beijing 100095, China; 3 School of Aeronautics, Northwestern Polytechnical University, Xi’an 710072, China; 4 AVIC Manufacturing Technology Institute Composite Technology Centre, Beijing 101300, China

**Keywords:** dispersion, high-strength glass fibre composites, hygrothermal environment, composite structure, mechanical property

## Abstract

High-strength glass fibre-reinforced composites (H-GFRPs) are widely used in various engineering fields because of their excellent mechanical properties and designability. The mechanical properties of H-GFRPs are more sensitive to temperature and humidity. Under high temperature and humidity conditions, the properties decrease greatly and the dispersion increases. Tensile, compressive, and in-plane shear tests were carried out on five batches of H-GFRPs under five different conditions, and the strength and stiffness properties under different test conditions were obtained. In this paper, the strength and stiffness properties of H-GFRPs under room temperature and hygrothermal conditions are statistically analysed based on macroscopic test data and the meso-bridging model. The results showed that under hygrothermal conditions, the dispersion of performance tended to decrease. The distribution types of other parameters are consistent with those under room temperature conditions, except for the transverse tensile modulus *E*_22,t_ and longitudinal compressive strength *X*_c_, which tend to follow a normal distribution. Among the four stiffness performance parameters, the correlation between *v*_12_ and the other three stiffness parameters was weak, whereas that between the other three stiffness parameters was strong.

## 1. Introduction

High-strength glass fibre-reinforced composites (H-GFRPs) are widely used in various engineering fields because of their excellent mechanical properties and designability. The complex manufacturing process of composite structures and the characteristics of integrated moulding of materials and structures result in the increased dispersion of the mechanical properties of composite structures [1,2]. Safety is a significant factor in the traditional deterministic design to ensure the security and reliability of composite structures. Owing to this inefficient and uneconomical design, the overall structure is overweight. More specifically, the advantages of composites with reduced size are significantly diminished. To fully determine the potential of composite materials and obtain an efficient composite structure design for ensuring the reliability and safety of structures, researchers have developed a probabilistic design method, which has been gradually applied to engineering structure design [3,4,5,6]. For example, in the late 20th century, the NASA Glenn Research Centre developed a method to simulate the probabilistic mechanical properties of composite structures, including micro-, meso-, and macro-mechanical property analysis modules [6]. Among the various factors that affect the mechanical properties of composite structures, the mechanical parameters of the corresponding material have the greatest impact on the overall structural bearing capacity. Therefore, a large dispersion of mechanical property parameters will also cause a large dispersion of the overall structural bearing capacity, which will threaten the safety and reliability of structural bearings. Therefore, it is necessary to analyse the dispersion of the basic mechanical properties of H-GFRPs to provide a reference for the engineering applications of this material.

At present, in engineering practice, the analysis and design of composite structures are generally performed based on the determined structural parameters, loads, and mechanical models. In deterministic design, the allowable load that the structure can bear is calculated according to the structural failure load obtained from strength analysis and a certain safety factor. However, several uncertainties exist in actual engineering structures that may cause large deviations in the structural response. Therefore, it is necessary to establish models that consider the uncertainties of the structural parameters and external loads. Currently, uncertainty models are mainly divided into probability, interval, and fuzzy models. After years of development, researchers have established a relatively perfect stochastic finite-element theory. Probability models based on stochastic theory mainly consider uncertainty based on the probability distribution characteristics of random parameters. By contrast, interval models examine uncertainty via interval analysis, for which only the upper and lower bounds of structural parameters need to be determined. Meanwhile, the fuzzy model primarily studies uncertainty based on the fuzzy statistical method. Among these three models, the probability model is the most widely used.

When evaluating the dispersion of the mechanical properties of composite structures, it is necessary to first evaluate the randomness of the mechanical properties of the composites. The existing research can be divided into two categories. The first is research based on experimental methods at the macro-scale. The second type is theoretical or numerical research based on the meso-scale. Generally, tests are performed on basic mechanical properties to determine the statistical characteristics of mechanical properties according to a large amount of test data and probability distribution models commonly used in statistics (normal distribution, lognormal distribution, two-parameter, or three-parameter Weibull distribution, etc.) [7,8,9,10,11]. For example, Jeong and Shenoi [7] conducted a series of tests on basic mechanical properties, obtained 35 test values for each material parameter, and determined the statistical characteristics of these material parameters by assuming that they follow a normal or Weibull distribution. The distribution parameters were fitted using test data. Zhao et al. [9] analysed the randomness of each performance parameter based on 15 test values according to the test results of the basic mechanical properties of unidirectional carbon fibre-reinforced composites. They used a normal distribution, lognormal distribution, and Weibull distribution to describe the randomness of these parameters. In addition, they studied the influence of different types of distributions on the randomness of composite structure strength and found that when the basic mechanical properties of materials obey a normal or lognormal distribution, the structural strength also obeys a normal or lognormal distribution, and its randomness is similar. However, when the strength properties of materials obey the Weibull distribution, the structural strength tends to follow a Weibull distribution.

In the absence or scarcity of test data, the distribution parameters of the mechanical properties are determined according to the composite manual or empirical assumptions. For example, Nakayama et al. [12,13] assumed that the transverse tensile and compression moduli, longitudinal compressive strength, and transverse compressive strength obeyed the normal distribution, wherein the expected value was the average value of the test, and the coefficient of variation (CV) was 0.05. In their study, Li et al. [14,15] assumed that the stiffness performance and ultimate strength of the composites obeyed a normal distribution, the expected value was the average value of the test, and the CV was assumed to be 0.05. In a subsequent study, they determined the CV according to the composite manual and found that the CV of each performance parameter differed in the range of 0.04–0.10 [16]. In addition, Li et al. [17] used a normal distribution to describe the randomness of four basic stiffness parameters, in which the expected value of the distribution parameters was determined according to the eigenvalues in the literature, and the CV was determined according to the composite manual and the literature. Mandal et al. [18] used a lognormal distribution to describe the randomness of the elastic modulus, shear modulus, and normal distribution to describe the randomness of Poisson’s ratio; however, they did not explain the source of the parameters of these two distributions. Meanwhile, Sepahvand and Marburg [19] proposed a method to estimate the randomness of the mechanical properties of composites based on limited test data. The theoretical or finite element simulation methods based on the meso-scale not only determine the statistical characteristics of mechanical properties of composites, but also obtain the correlation between the properties’ parameters. Shaw, etc. [20] calculated the mechanics performance of the composites based on the bridging model proposed by Zhengming Huang. The component material properties were treated as random input variables, random sampling was carried out according to the Monte Carlo sampling method, and then the randomness of the mechanical properties of composite materials was determined. Toft et al. [21] constructed a meso-mechanics theoretical model combined with the Monte Carlo method and predicted the statistical characteristics of glass fibre-reinforced composites. Alazwari and Rao [22] used the meso-mechanics theoretical model [23] proposed by Chamis to predict the uncertainty of stiffness properties of composite materials. The multi-scale analysis methods were used to predict the stiffness of the material properties and distribution characteristics; in the microscopic scale, the RVE structure unit with the random distribution of components material volume was considered, while for the mechanical properties of materials, the mesoscopic scale was used, and then the probability of its stiffness performance and relevance was analysed [24,25,26].

Accordingly, in this study, the dispersion of the mechanical properties of different batches of H-GFRPs was analysed based on the test data. A probability distribution analysis method for mechanical properties based on macro test data and a meso-mechanical model was proposed, and the probability distribution characteristics of the mechanical properties at room temperature (RT), in a dry state, and in a hygrothermal environment were determined. Subsequently, the reliability of the material mechanical properties based on the deterministic and probabilistic design methods was analysed.

## 2. Dispersion Analysis of Mechanical Properties of Different Batches of High-Strength Glass Fibre-Reinforced Composites (H-GFRPs)

### 2.1. Experimental Program

In this paper, the specimens were provided by China Composite Group Corporation Ltd. (AVIC COMPOSITE CO., LTD, Beijing, China), which were manufactured from laminates made of H-GFRP (AC318/S_6_C_10_-800). Five test environmental conditions were designed as shown in Table 1. The hygrothermal condition is 70 °C and 85% RH, and the equilibrium is achieved when the average mass of travellers changes less than 0.05% for two consecutive readings within a span of 7 days.

According to standard ASTM D3039, D6641, and D3518, the tensile, compression, and plane shear tests of H-GFRP in five environments were carried out [27,28,29], and the test types and performance parameters are shown in Table 2. The bi-axial average extensometer was used to measure the specimen deformation in the tensile test. In the compression test, a strain gauge is bonded on the gage section of the sample to measure the deformation. The axial and transverse extensometers were used to measure the axial and transverse deformation of the specimen in the plane shear test. Under conditions I and III, when the temperature of the specimen reached the test temperature, it was held for 5 min before the mechanical test was carried out. Under conditions IV and V, when the temperature of the specimen reached the test temperature, it was held for 2 min before the mechanical test was carried out.

According to the stable batch mechanical property test of AC318/S_6_C_10_-800 composites, we can obtain the basic mechanical parameters of H-GFRP from five types of tests, including six stiffness properties and five strength properties, as detailed in Table 1.

It is worth noting that the H-GFRPs considered in the test came from five batches, as shown in Table 3. To evaluate the stability of the material properties produced in different batches, the respective measured values were analysed for dispersion.

### 2.2. Experimental Results

Table 4 gives the average value, standard deviation, and coefficient of variation of the stiffness properties of the five batches of H-GFRPs under the different conditions. The results show that under conditions IV and V, the stiffness properties controlled by the fibre, such as *E*_11,t_ and *E*_11,c_, basically do not change while the properties controlled by the matrix, such as *E*_22,t_, *E*_22,c_ and *G*_12_, decrease greatly.

In order to quantitatively measure the stability of the strength properties of each batch of materials, the average value, standard deviation and coefficient of variation of the strength properties of five batches of H-GFRP are given in Table 5. It can be seen that the coefficient of variation of most strength performance data is less than 10%. The results showed that under conditions IV and V, the strength controlled by both the fibre and matrix decreased significantly, especially at high humidity, decreasing 50% compared to condition II, indicating that glass fibre is more sensitive to humidity.

### 2.3. Dispersion Analysis of Stiffness Performance

**Figure 1:**
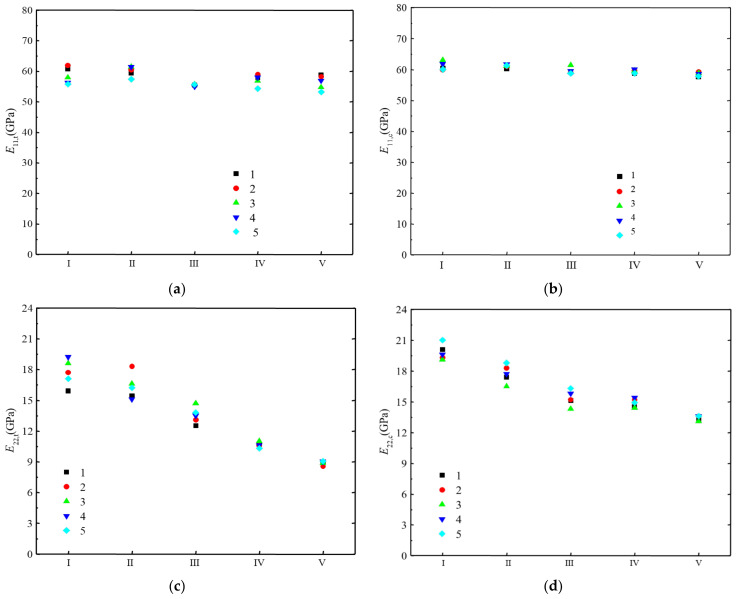

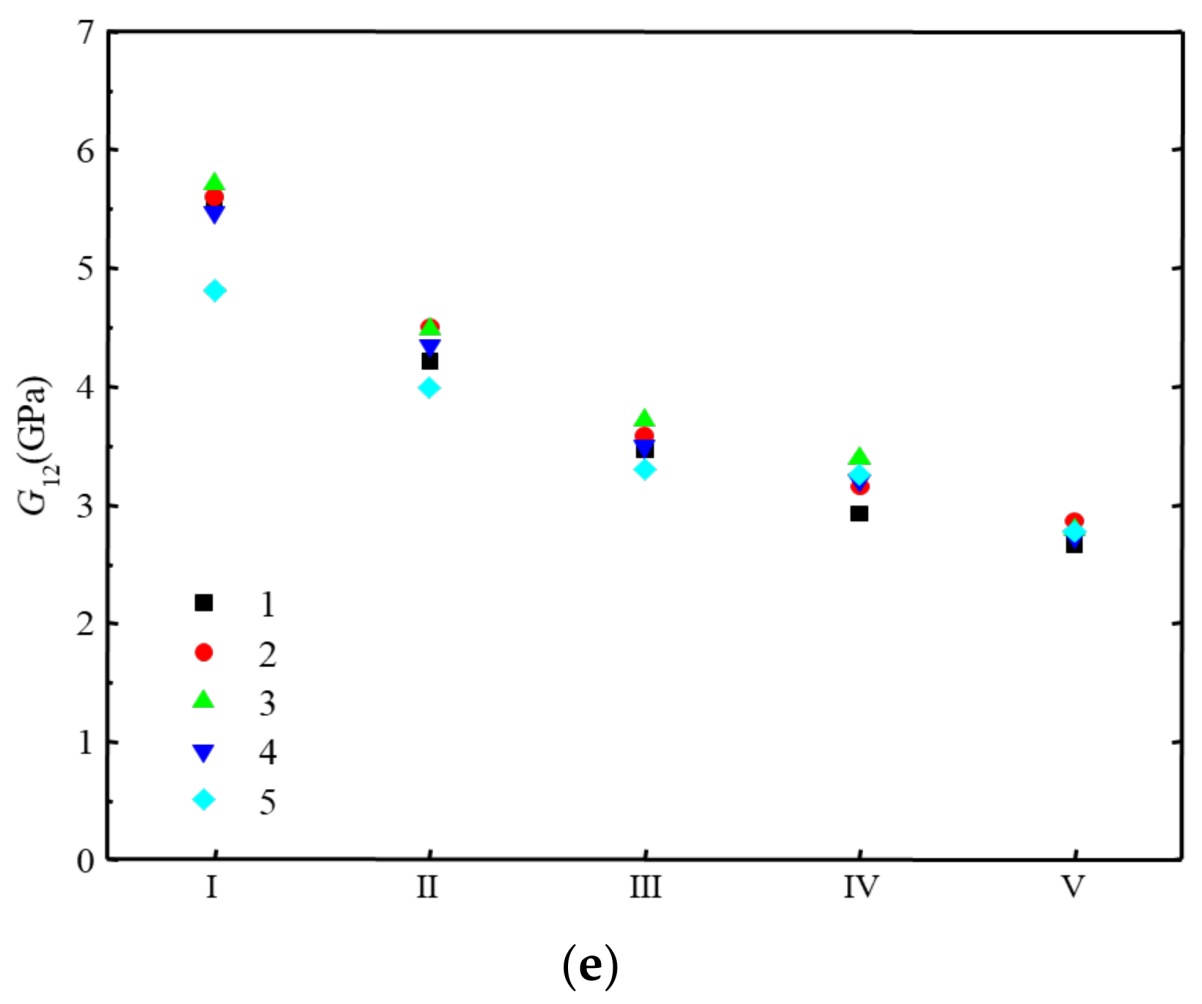
Test results of stiffness performance of H-GFRPs. (**a**) *E*_11,t_, (**b**) *E*_11,c,_ (**c**) *E*_22,t_, (**d**) *E*_22,c,_ (**e**) *G*_12_.

Figure 1 presents the stiffness performance data for the five batches of H-GFRPs under five humid and hot working conditions. Each data point in the figure represents the average value of the test results for the six samples. As can be seen, the data dispersion is generally small, indicating that the material performance stability of the five batches is high, and besides the longitudinal tensile–compression modulus, the dispersion between batches decreases with the deterioration of the humid and hot conditions.

In probability theory and statistics, the CV, also known as the dispersion coefficient, is a normalised measure of the dispersion degree of the probability distribution. The CV has been defined as the ratio of the standard deviation to the average value. The CV can eliminate the unit sum (or) of the influence of different averages on the comparison of the variation degree of two or more data points. Therefore, we used the CV to measure the stability of the mechanical properties of the different batches of materials.

**Figure 2:**
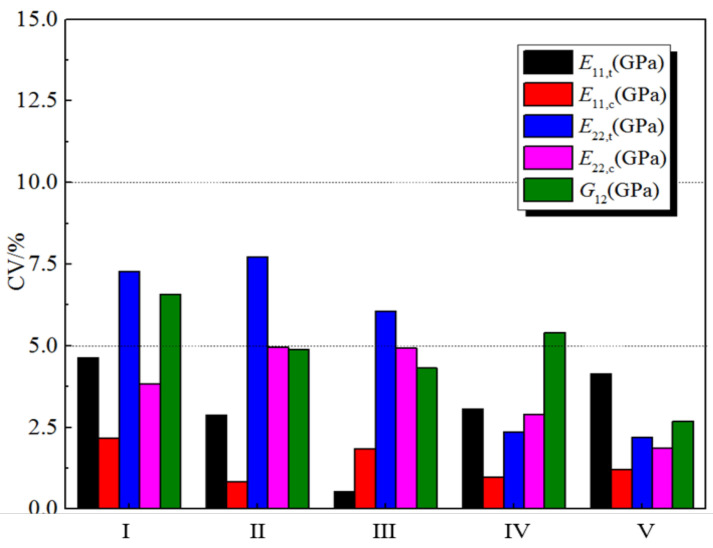
Coefficient of variation (CV) for the stiffness properties of the five H-GFRP batches.

Figure 2 displays the CV results of the five stiffness properties under the five humid and hot working conditions. As can be seen, the CV values are mostly less than 5%. We divided the CV results into three categories: 0–5%, 5–10%, and 10–15%. Among the 25 groups of test results, the CV of 80% was less than 5%, and that of 20% was between 5% and 10%. Generally, in the statistical analysis of data, CV < 5% indicates good data stability. Therefore, we can conclude that although the H-GFRPs originated from different batches, the stiffness performance stability of each batch is good.

**Figure 3:**
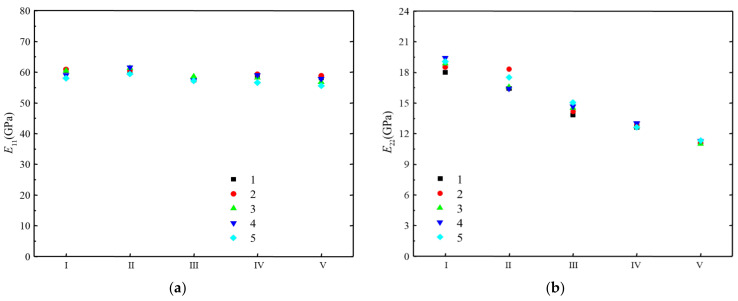
Longitudinal and transverse tensile–compression moduli of the five H-GFRP batches. (**a**) *E*_11_, (**b**) *E*_22_.

In addition, in the mechanical analysis of composite structures, the tensile and compression moduli are often not distinguished because they are usually very close. It can also be seen from the test results that the tensile and compression moduli of the H-GFRPs are similar. Therefore, we averaged the tensile and compression moduli of each batch of materials to obtain the longitudinal tensile–compression modulus *E*_11_ and transverse tensile–compression modulus *E*_22_, as shown in Figure 3. It can be seen that the dispersion of the longitudinal tensile–compression modulus is very small, and is equivalent under different humid and hot conditions. More precisely, the dispersion of the transverse tensile–compression modulus decreases with the increasing severity of the humid and hot conditions.

Table 6 lists the average values, standard deviations, and CVs of the longitudinal and transverse tensile–compression moduli of the five H-GFRP batches. It is apparent that the CVs of the longitudinal and transverse tensile–compression moduli are not more than 2.1% and 5%, respectively. Therefore, the longitudinal and transverse tensile–compression moduli of the different H-GFRP batches are stable.

### 2.4. Dispersion Analysis of Strength Properties

**Figure 4:**
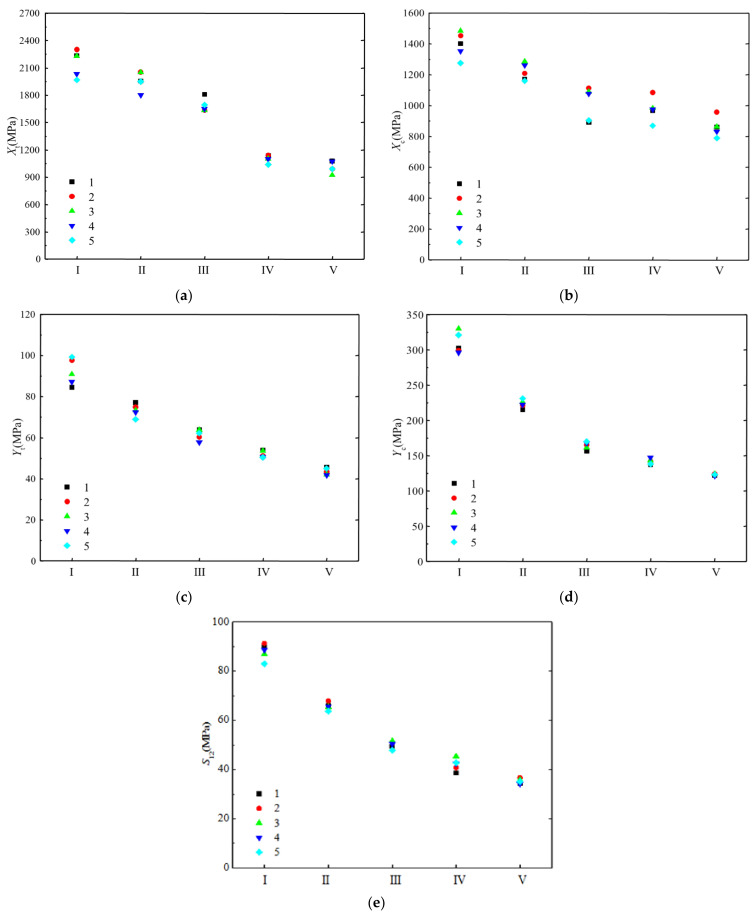
Test results of the strength properties of H-GFRPs. (**a**) *X*_t_, (**b**) Xc, (**c**) *Y*_t_, (**d**) *Y*_c_, (**e**) *S*_12_.

Figure 4 displays the strength performance data of the five H-GFRP batches under five humid and hot working conditions. Each data point in the figure represents the average value of the test results for the six samples. It can be seen that the longitudinal tensile and compressive strengths of the five batches of materials have a certain dispersion, which is equivalent under the five humid and hot working conditions. The dispersions of transverse tensile strength, transverse compressive strength, and the longitudinal and transverse shear strengths are small, and decrease with diminishing heat and humidity conditions.

**Figure 5:**
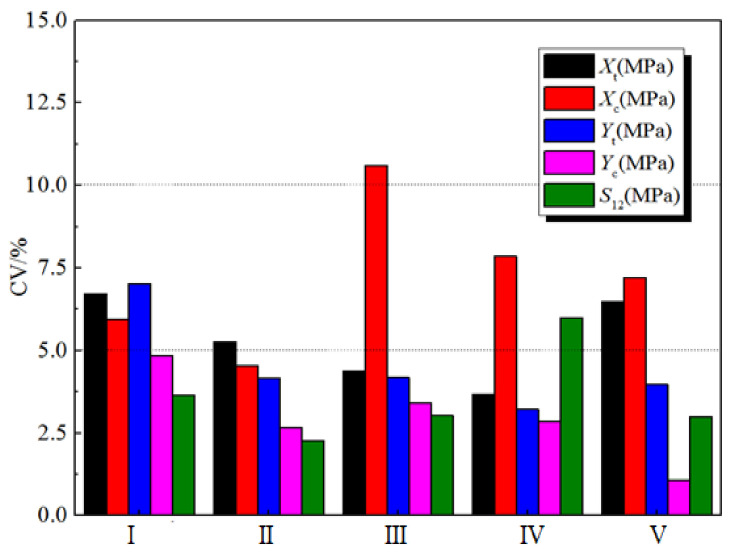
CV results for the strength properties of the five H-GFRP batches.

Figure 5 depicts the CV results for the five strength properties under the five humid and hot working conditions. In the 25 groups of data, 64% of the CV values are lower than 5%, 32% are between 5% and 10%, and 4% are between 10% and 15%. Among the five strength parameters, the CVs of the longitudinal compressive strength and longitudinal tensile strength were the largest. In the five humid and hot working conditions, the CV in the RTD state was smaller than that in the other four working conditions, indicating that the stability of the strength properties between batches was best in the RTD state.

In general, the strength performance dispersion of the five H-GFRP batches was greater than that of the stiffness performance. By comparing and analysing the CV of the test results of the five batches, it can be concluded that the stiffness performance stability of each batch of materials is good, and the strength performance stability is moderate.

## 3. Random Analysis of the Mechanical Properties of High-Strength Glass Fibre Composites

### 3.1. Determination of the Probability Distribution of Material Mechanical Properties Based on Macro Test Data

#### 3.1.1. Methods

Existing literature also shows that the randomness of the mechanical properties of composites can be determined based on macro test data. The analytical process for this method was conducted as follows.

First, Origin^®^ Software or other statistical analysis software was used to determine several test values of basic mechanical property parameters, wherein appropriate intervals were selected for frequency statistics to obtain the corresponding cumulative frequency for calculating the cumulative probability distribution.

Second, three widely used continuous probability distribution functions, normal distribution, lognormal distribution, and Weibull distribution, were used for fitting, and their cumulative distribution functions (CDFs) are given by:

(1) $$\begin{array}{l} F(x)\left|{}_{Normal}\right.=\frac{1}{2}\left[1+\mathrm{erf}\left(\frac{x-\mu }{\sqrt{2}\sigma }\right)\right]\\ F(x)\left|{}_{Lognormal}\right.=\frac{1}{2}\left[1+\mathrm{erf}\left(\frac{\ln x-\mu }{\sqrt{2}\sigma }\right)\right]\\ F(x)\left|{}_{Weibull}\right.=1-\exp\left[-{\left(\frac{x}{\lambda }\right)}^{\kappa }\right] \end{array}$$

where the error function is defined as $\mathrm{erf}(x)=\frac{2}{\sqrt{\pi }}\int_{0}^{x}e^{-t^{2}}dt$. In terms of the data fitting methods, the rationality of the fitting results can be judged by the goodness-of-fit. Generally, the parameter value representing goodness of fit varies from 0 to 1. The closer the value is to 1, the better fitting the model. Therefore, the best distribution corresponding to each mechanical property parameter can be determined by comparing the goodness-of-fit of the three distributions.

Finally, a histogram of the mechanical property parameters was obtained using a statistical method, and the probability density function of the optimal distribution determined above is drawn in the figure for verification.

#### 3.1.2. Probability Distribution Analysis of the Mechanical Properties of Materials under the Room Temperature and Dry (RTD) Condition

(1) Probability distribution characteristics of the stiffness properties in the RTD state

**Figure 6:**
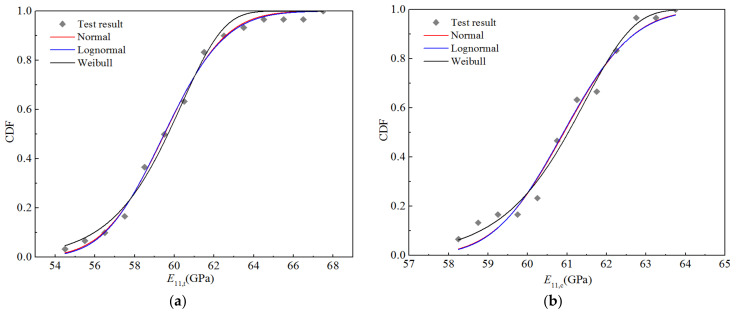

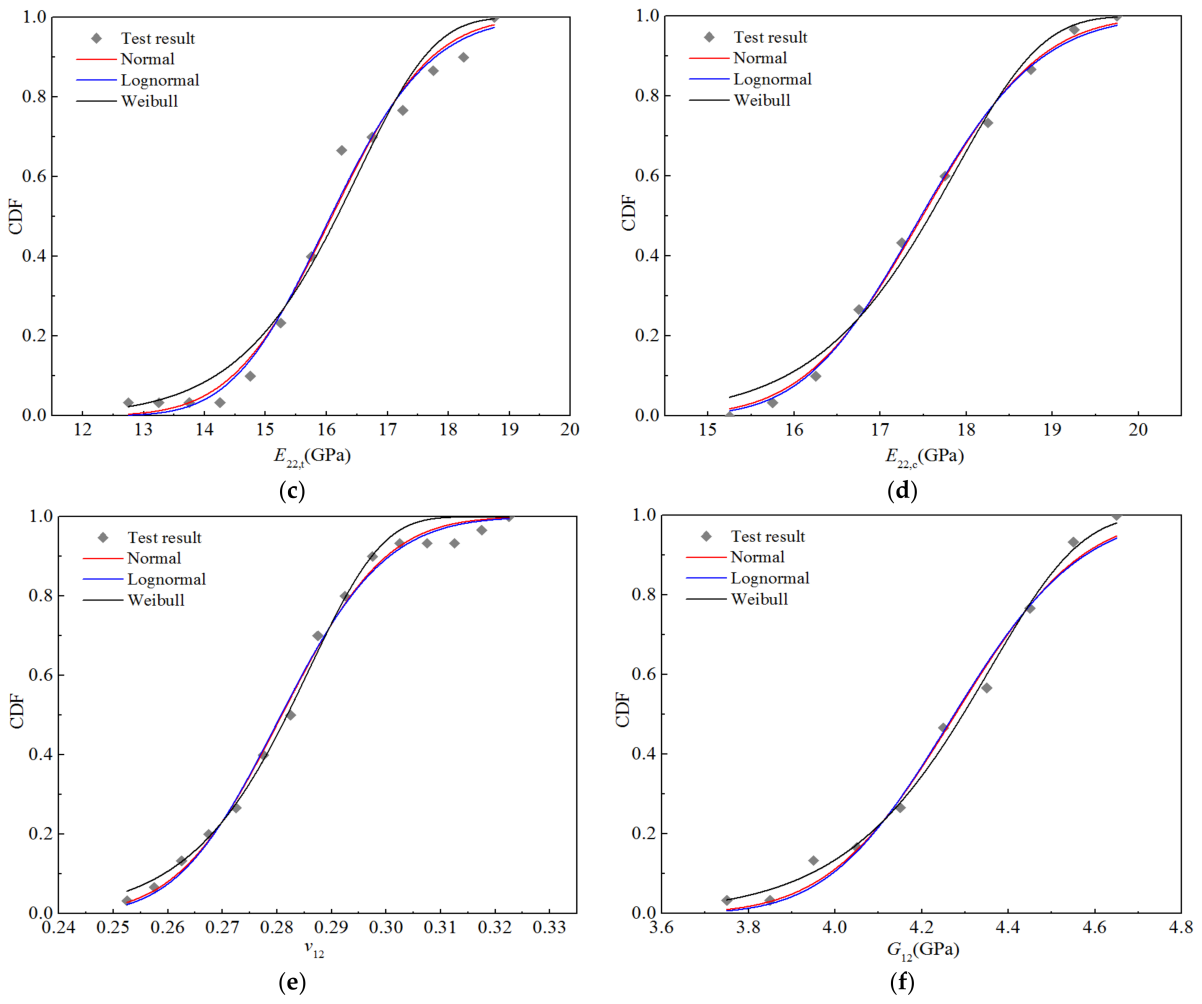
Fitting curves for the stiffness properties of H-GFRPs at RT. (**a**) *E*_11,t_, (**b**) *E*_11,c_, (**c**) *E*_22,t_, (**d**) *E*_22,c,_ (**e**) *v*_12_, (**f**) *G*_12_.

Using the above method, 30 test values based on stiffness performance for the RTD condition (five batches in total, six pieces in each batch) were used to calculate the cumulative probability distribution, as depicted in Figure 6; the normal, lognormal, and Weibull distributions were used for fitting, and the fitting curves are displayed in Figure 6. It can be seen that the fitting curves of the normal and lognormal distributions almost coincide, indicating that the randomness of the stiffness performances described by these two distributions are essentially equivalent, while the upper and lower tails of the Boolean distribution curve obviously differ from the two distributions mentioned above. Nevertheless, as shown in Figure 6, the three fitting curves are generally in good agreement with the test data points.

Table 7 lists the distribution parameters of the three distributions corresponding to the fitting curves, and the parameters representing the goodness-of-fit. We believe that the distribution with goodness of fit closest to 1 is the optimal distribution. It can be seen that the longitudinal tensile modulus *E*_11,t_, transverse tensile modulus *E*_22,t_, and transverse compression modulus *E*_22,c_ tend to obey lognormal distribution, the in-plane Poisson’s ratio *v_12_* tends to obey the normal distribution, while the longitudinal compression modulus *E*_11,c_ and longitudinal and transverse shear modulus *G*_12_ tend to obey the Weibull distribution. It is worth noting that the goodness-of-fit values for the normal and lognormal distributions are very close, again demonstrating the equivalence of these two distributions in describing the randomness of material mechanical properties. In addition, the goodness-of-fit for the best distribution of *E*_11,c_ and *E*_22,t_ is 0.986, while those for the optimal distributions of other parameters are 0.995 or above.

**Figure 7:**
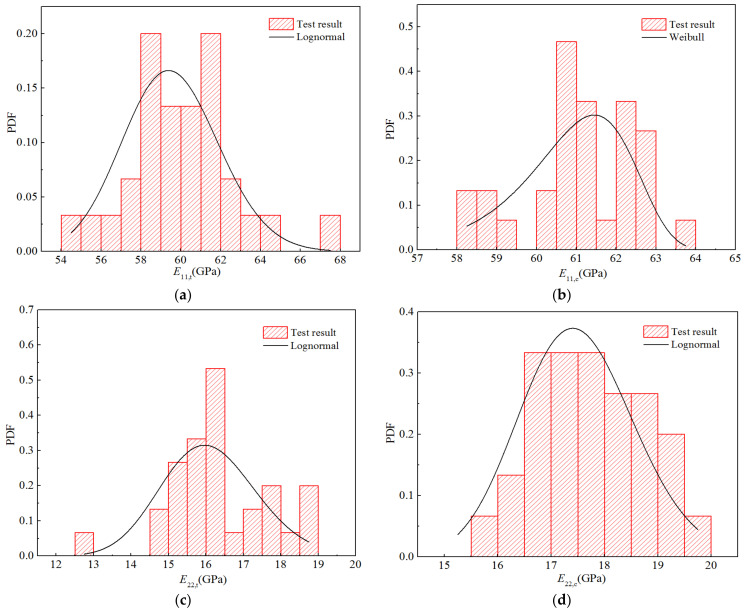

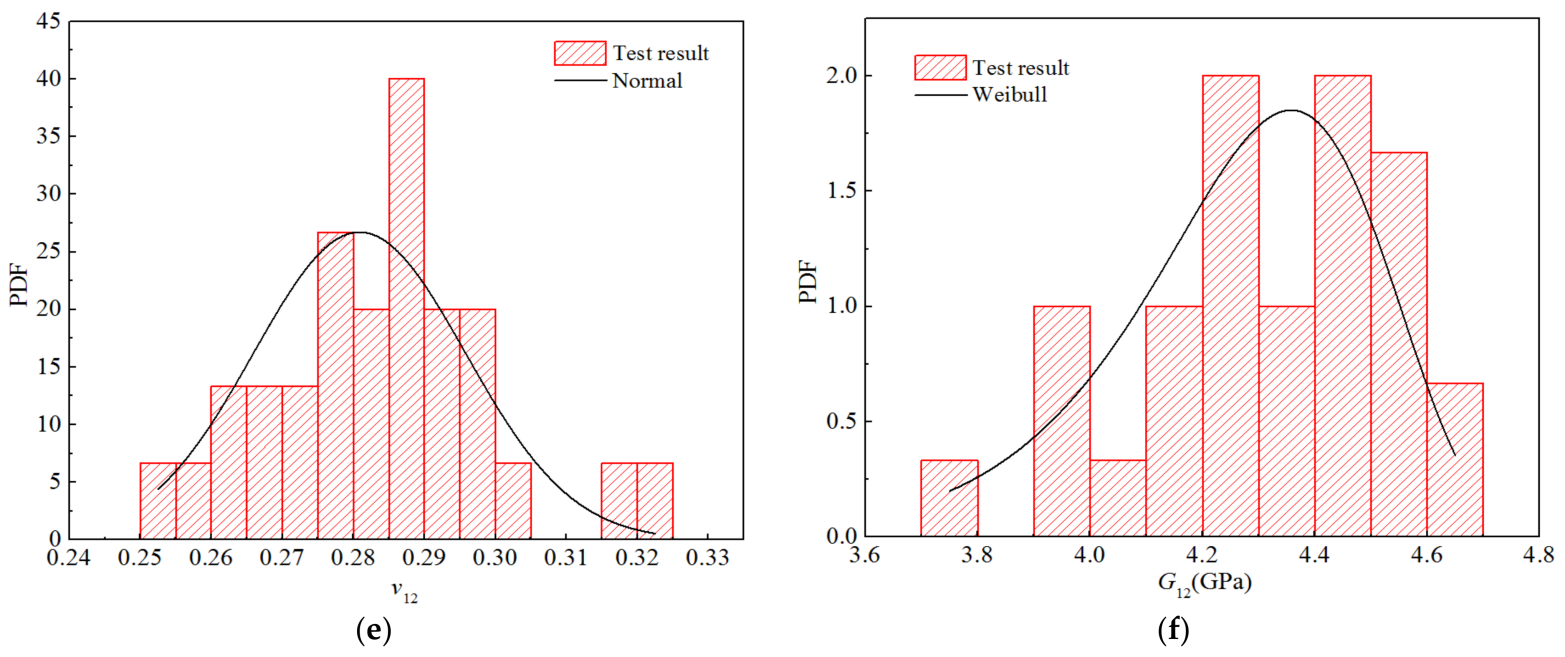
Probability function diagrams for the stiffness properties of H-GFRPs at RT. (**a**) *E*_11,t_, (**b**) *E*_11,c,_ (**c**) *E*_22,t_, (**d**) *E*_22,c,_ (**e**) *v*_12_, (**f**) *G*_12_.

Furthermore, based on 30 test values, five histograms for the stiffness properties were obtained using a statistical method, and the probability density functions of the optimal distributions determined above are depicted in Figure 7. Owing to the small amount of test data, the statistical histograms contain breakpoints and cannot accurately reflect the parameter distribution characteristics. However, it can be observed that the resulting statistical histograms closely correspond to the trends of the optimal distributions.

(2) Probability distribution characteristics of the strength properties in the RTD state

**Figure 8:**
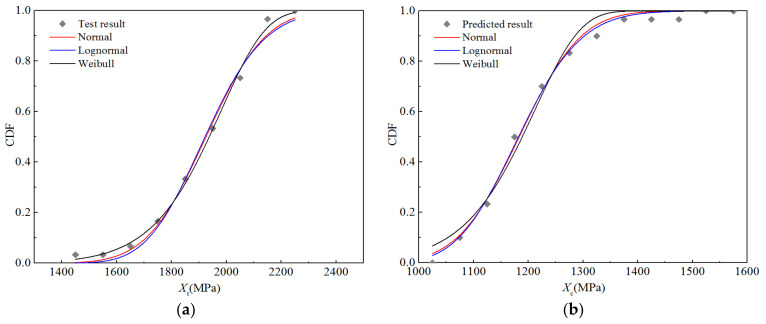

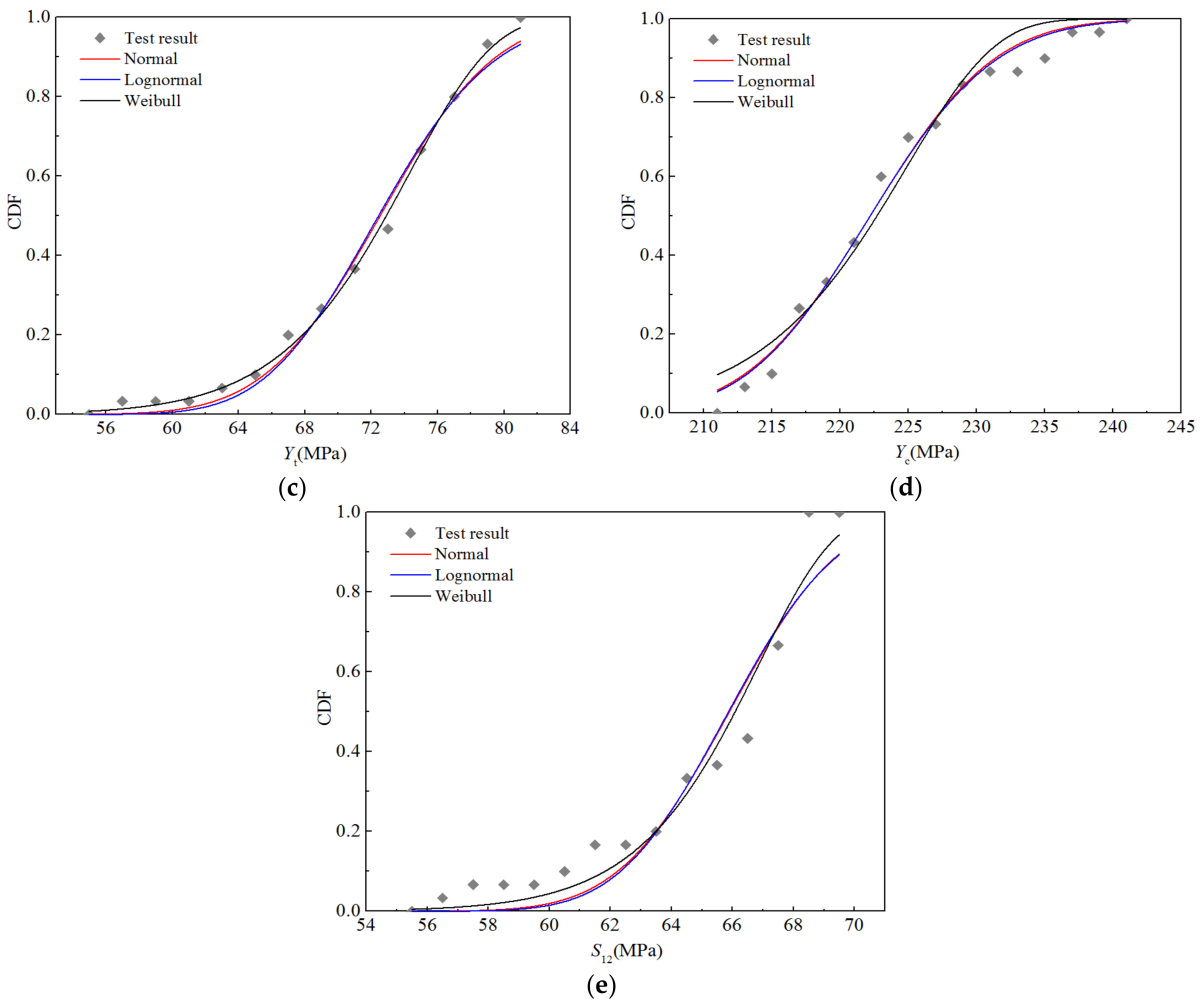
Fitting curves for the strength performance functions of H-GFRPs at RT. (**a**) *X*_t_, (**b**) *X*_c,_ (**c**) *Y*_t_, (**d**) *Y*_c,_ (**e**) *S*_12_.

Figure 8 displays the cumulative distribution probabilities of the five strength properties, as well as the fitting curves of the three distributions under the RTD condition. It is apparent that the fitting curves of the normal and lognormal distributions almost coincide, and there are only slight differences between the upper and lower tails, whereas the Weibull distribution curve is significantly different from the first two. Nevertheless, it can be seen that the three fitting curves are in good agreement with the test data points.

Table 8 lists the parameters of the three distributions corresponding to the fitting curve and those representing the goodness-of-fit. The optimal distribution of the three distributions was determined according to the goodness-of-fit value. It can be seen that the longitudinal compressive strength *X*_c_ and transverse compressive strength *Y*_c_ tend to obey the lognormal distribution, while the longitudinal tensile strength *X*_t_, transverse tensile strength *Y*_t_, and longitudinal and transverse shear strength *S*_12_ tend to obey the Weibull distribution. In addition, regarding the optimal distribution, although the goodness-of-fit values for *Y*_c_ and *S*_12_ are 0.987 and 0.955, respectively, those for the other three strength performance parameters are higher than 0.995.

**Figure 9:**
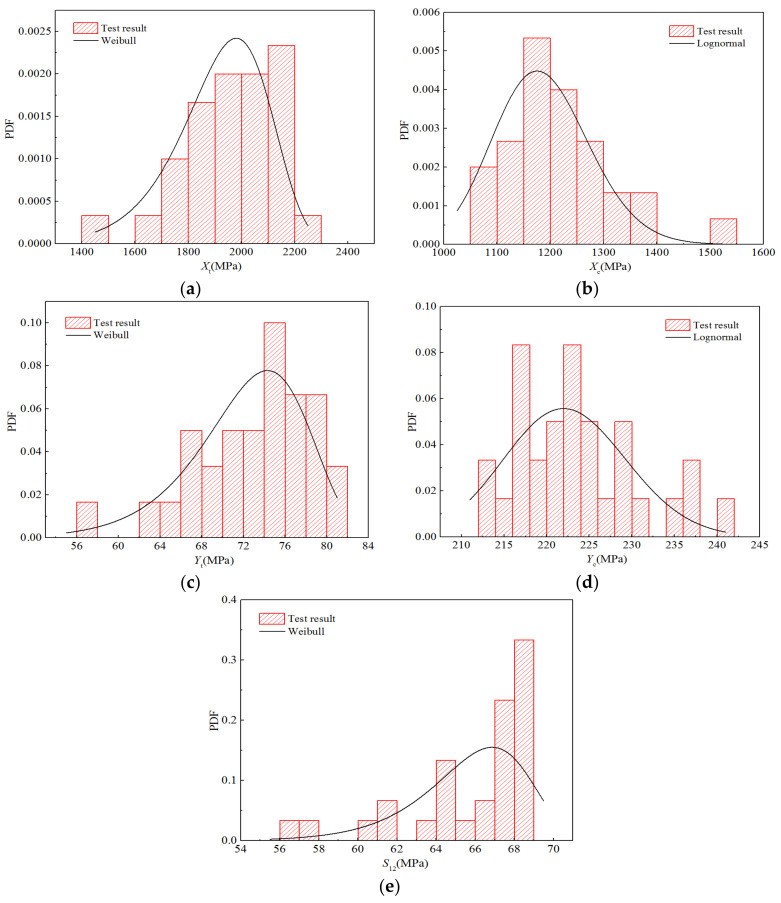
Probability distribution diagrams for the strength properties of H-GFRPs under the RTD condition. (**a**) *X*_t_, (**b**) *X*_c,_ (**c**) *Y*_t_, (**d**) *Y*_c,_ (**e**) *S*_12_.

Moreover, Figure 9 displays five histograms for the intensity performance and probability density functions of the optimal distribution determined above. Although breakpoints occur in the histograms owing to the limited test data, it can be seen that the distribution trends of the histograms obtained using statistics closely correspond to that of the optimal distribution.

#### 3.1.3. Probability Distribution Analysis of the Mechanical Properties of Materials under the ETW Condition

Among the five environmental working conditions, except for RTD, condition V was the worst working condition, i.e., at 70 °C high temperature and equilibrium moisture absorption, as described in Table 2. It can further be seen from the test results that the high temperature of 70 °C and equilibrium moisture absorption conditions degrade the mechanical properties of H-GFRPs the most. Therefore, the method described in Section 3.1.1 was used to analyse the probability distribution characteristics of the mechanical properties of the materials under this condition.

(1) Probability distribution characteristics of the stiffness performance in a humid and hot environment

**Figure 10:**
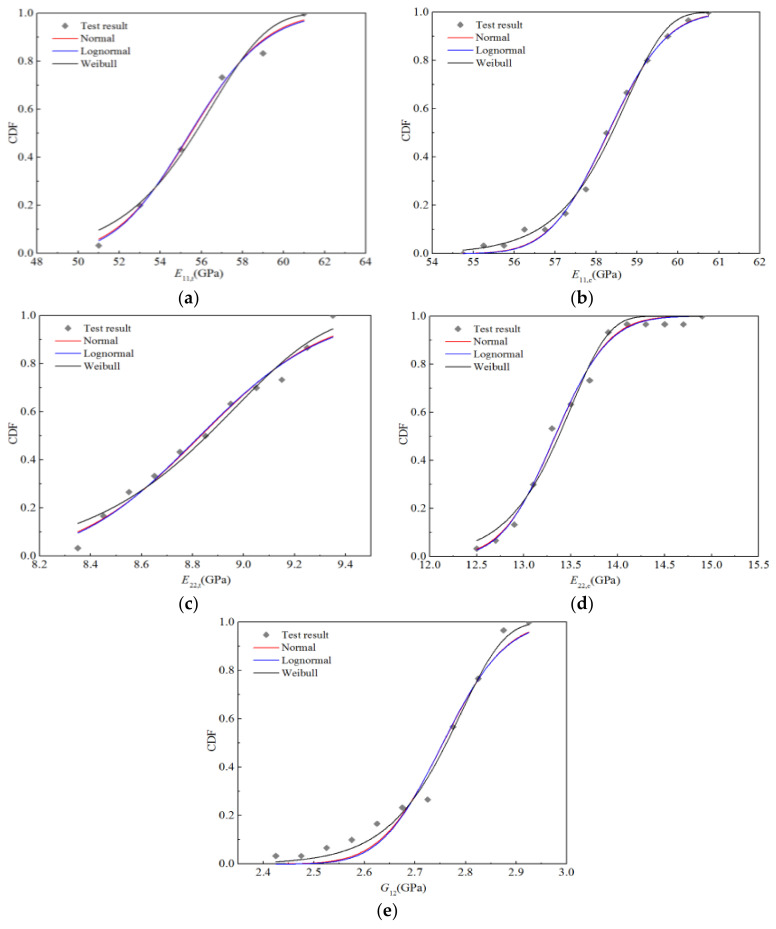
Functional fitting curves for the strength properties of H-GFRPs in a humid and hot environment. (**a**) *E*_11,t_, (**b**) *E*_11,c,_ (**c**) *E*_22,t_, (**d**) *E*_22,c,_ (**e**) *G*_12_.

Figure 10 exhibits the cumulative distribution probabilities for the five stiffness properties in addition to the fitting curves of the three distributions under a humid and hot environment. It can be seen that the fitting curves for the normal and lognormal distributions almost coincide, whereas that for the Weibull distribution is clearly different from the first two. However, in general, the fitting curves of these three distributions are in good agreement with the experimental data points.

Table 9 lists the distribution parameters representing the goodness-of-fit corresponding to the three distribution types and their fitting curves. The best distribution was determined according to the goodness-of-fit value. It can be seen that the longitudinal tensile modulus *E*_11,t_ and transverse compression modulus *E*_22,c_ tend to obey the lognormal distribution, the transverse tensile modulus *E*_22,t_ tends to obey the normal distribution, while the longitudinal compression modulus *E*_11,c_ and longitudinal and transverse shear modulus *G*_12_ tend to obey the Weibull distribution. Although the goodness-of-fit values of the best distribution for *E*_22,t_ and *G*_12_ are 0.976 and 0.988, respectively, those for the other parameters are higher than 0.99.

**Figure 11:**
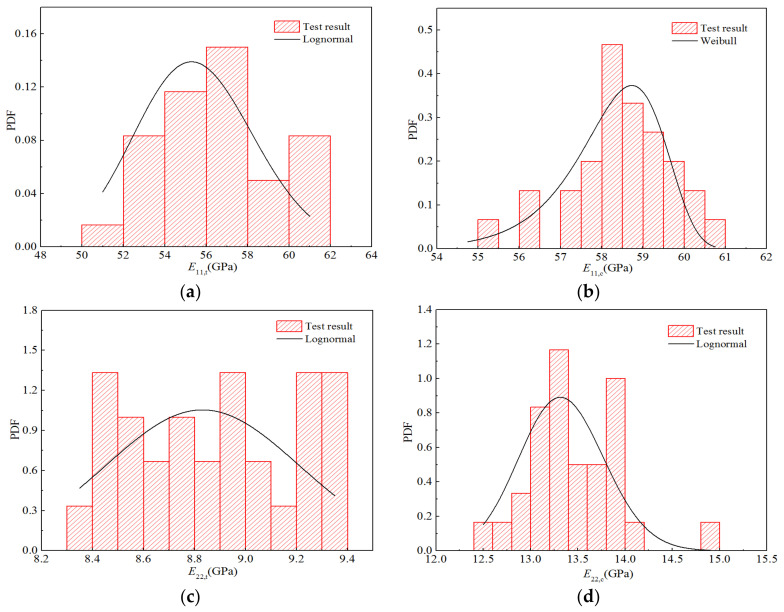

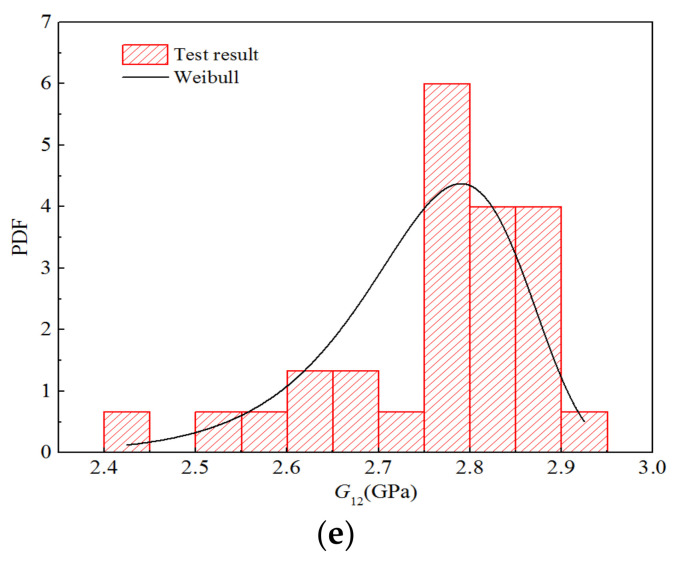
Probability distribution diagrams for the strength properties of H-GFRPs under the high-temperature equilibrium moisture absorption (ETW) condition. (**a**) *E*_11,t_, (**b**) *E*_11,c,_ (**c**) *E*_22,t_, (**d**) *E*_22,c,_ (**e**) *G*_12_.

Figure 11 displays the obtained statistical histograms for the five stiffness properties as well as the probability density function of the optimal distribution under a humid and hot environment. The histograms of all the parameters, except that for the transverse tensile modulus *E*_22,t_, are consistent with the distribution trends.

(2) Probability distribution characteristics of the strength properties in a humid and hot environment

**Figure 12:**
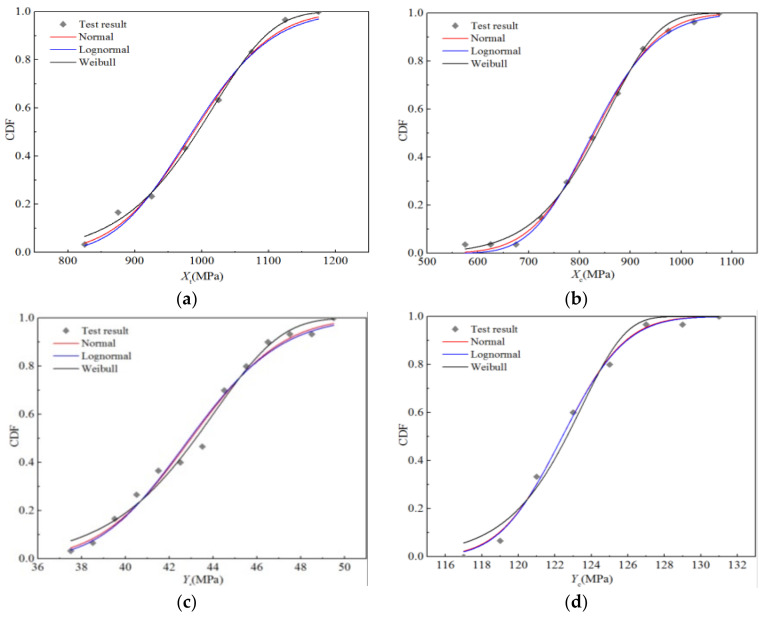

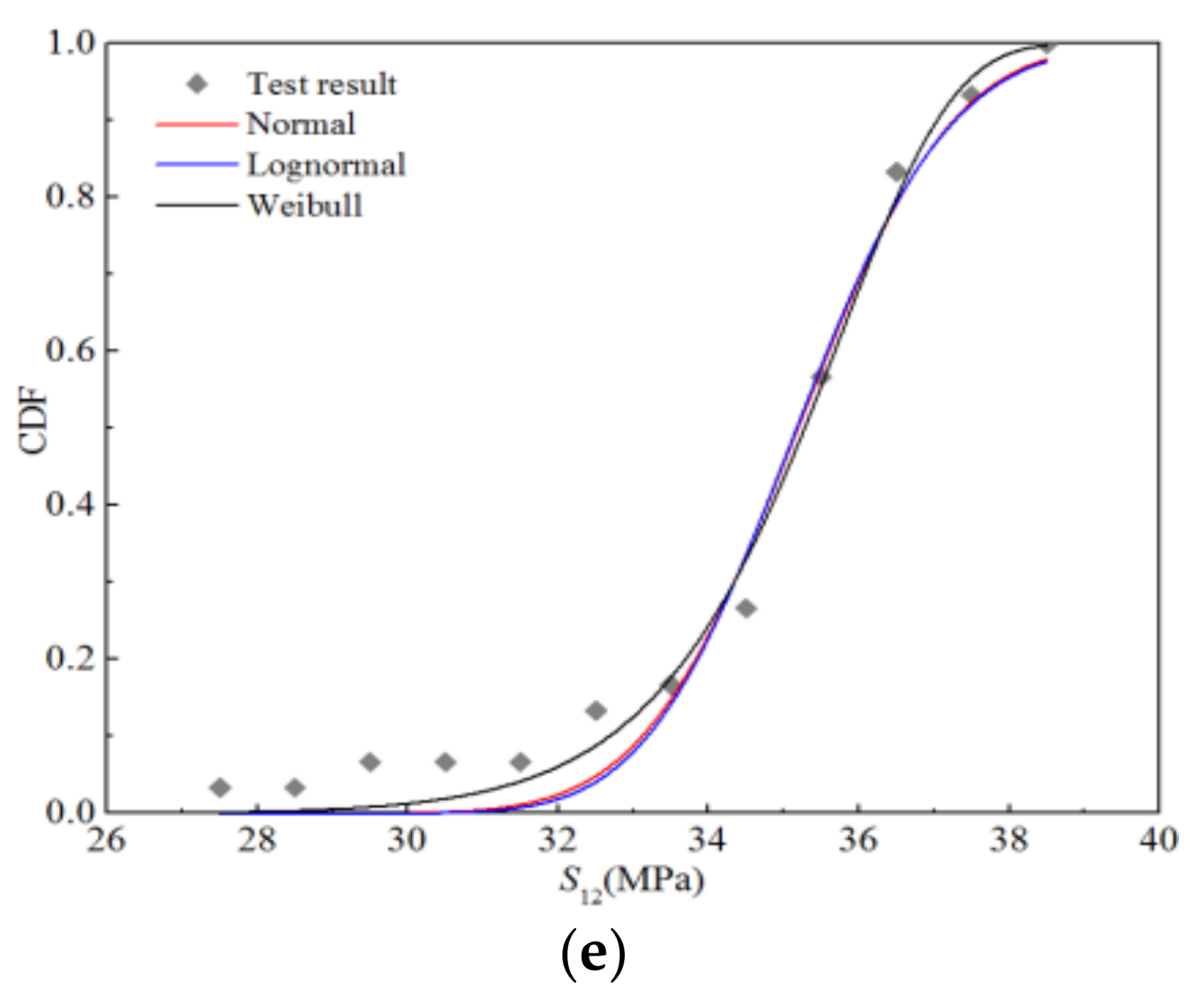
Functional fitting curves for the strength properties of H-GFRPs under the ETW condition. (**a**) *X*_t_, (**b**) *X*_c,_ (**c**) *Y*_t_, (**d**) *Y*_c,_ (**e**) *S*_12_.

Figure 12 presents the cumulative probability distribution of the five strength performance parameters and the fitting curves of the three distributions under humid and hot environment. It is clear that the fitting curves of the normal and lognormal distributions almost coincide, and there is only a slight difference between the upper and lower tails, whereas the Weibull distribution curve is significantly different from the first two. However, the fitting curves of these three distributions are in good agreement with the experimental data.

Table 10 shows the distribution parameters of the three distributions corresponding to the fitting curve, and the parameters representing the goodness-of-fit. The best distribution was determined according to the goodness-of-fit value. It can be seen that the longitudinal compressive strength *X*_c_ tends to obey the normal distribution, the transverse compressive strength Y_c_ tends to obey the lognormal distribution, and the longitudinal tensile strength *X*_t_, transverse tensile strength Y_t_, and longitudinal and transverse shear strength S_12_ tend to obey the Weibull distribution. The goodness-of-fit values of the best distribution for transverse tensile strength Y_t_ and longitudinal and transverse shear strength S_12_ are 0.987 and 0.989, respectively, whereas that for the other parameters is higher than 0.995.

**Figure 13:**
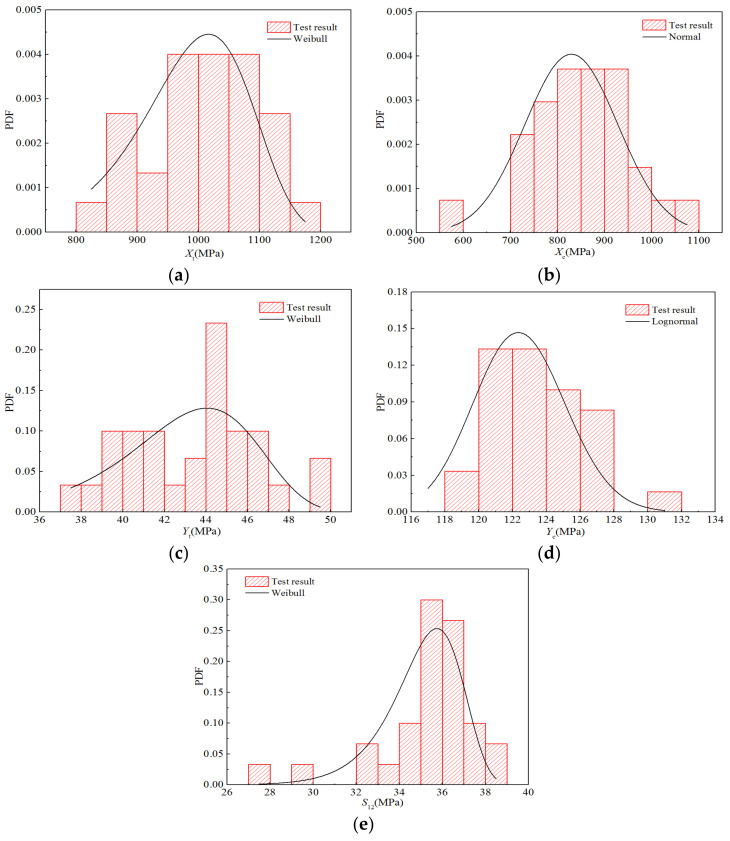
Probability distribution diagrams for the strength properties of H-GFRPs under the ETW condition. (**a**) *X*_t_, (**b**) *X*_c,_ (**c**) *Y*_t_, (**d**) *Y*_c,_ (**e**) *S*_12_.

In addition, statistical histograms for the five strength performance parameters in a humid and hot environment were realized using a statistical method and are displayed in Figure 13, including the probability density function of the optimal distribution. It can be seen from this figure that although some histograms are discontinuous owing to limited test data, they have trends similar to those of the optimal distributions.

#### 3.1.4. Comparison of the Probability Distribution Characteristics of the Mechanical Properties under the RTD and ETW Conditions

(1) Comparison of the probability distribution characteristics of stiffness performance

Table 11 presents the RTD and humid environment details, probability distribution types, and distribution parameters corresponding to stiffness performance under the ETW condition (70 °C high temperature and equilibrium moisture absorption). It can be seen that although the transverse tensile modulus *E*_22,t_ obeys the lognormal distribution in the RTD state and normal distribution in the humid and hot environment, the distribution types of the other parameters under the two working conditions are consistent. According to the previous analysis, we know that the lognormal and normal distributions are equivalent in describing the statistical characteristics of the material mechanical properties. It can be seen that humid and hot environments do not cause changes in the probability distribution type of the stiffness properties of the H-GFRPs.

**Figure 14:**
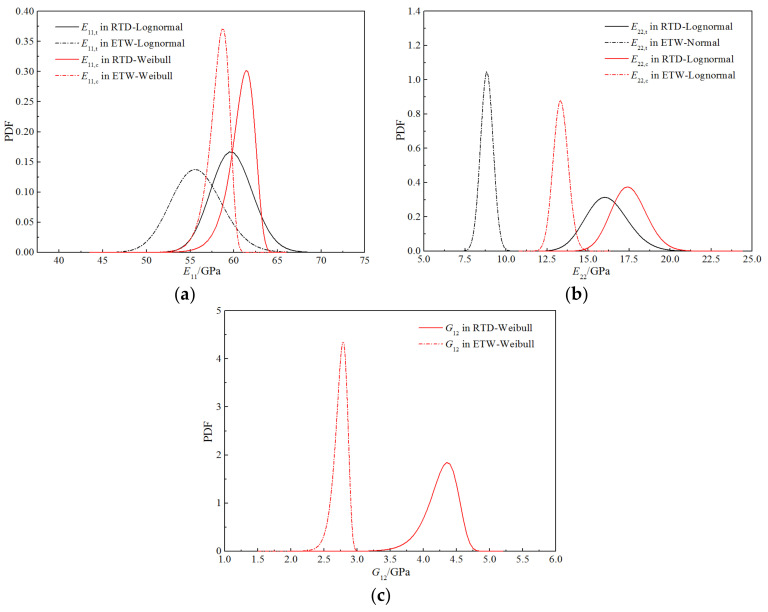
Comparison of the probability curves for the stiffness properties of H-GFRPs. (**a**) *E*_11_, (**b**) *E*_22,_ (**c**) *G*_12_.

Figure 14 depicts the probability density functions for the stiffness performance of the H-GFRPs under the two working conditions. It can be seen that the peak positions of the curves for the longitudinal tensile and longitudinal compression moduli under the two working conditions are relatively close, and the curve shapes are similar; thus, the humid and hot environment conditions have little influence on the probability distribution characteristics of these two parameters. The curves for the transverse tensile, transverse compression, and longitudinal and transverse shear moduli under the two working conditions have not only significantly different peak positions, but also different curve shapes. Thus, the humid and hot environment conditions significantly influence these three parameters.

(2) Probability distribution characteristics of the strength properties under the humid and hot environment conditions

Table 12 presents the probability distribution types and corresponding parameters for strength performance under the RTD and humid environment conditions (high temperature of 70 °C and equilibrium moisture absorption). It can be seen that the distribution types of all parameters are consistent under the two working conditions, except that for the longitudinal compressive strength *X*_c_, which obeys a lognormal distribution in the RTD state and a normal distribution in the humid and hot environment. According to the previous analysis, we know that the lognormal and normal distributions are similar, and the mechanical property parameters of the materials are basically equivalent in terms of statistical characteristics. It can be observed that the humid and hot environment conditions do not change the probability distribution type corresponding to the strength properties of the H-GFRPs.

**Figure 15:**
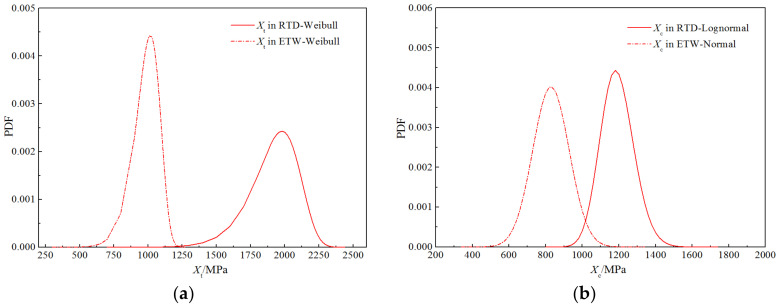

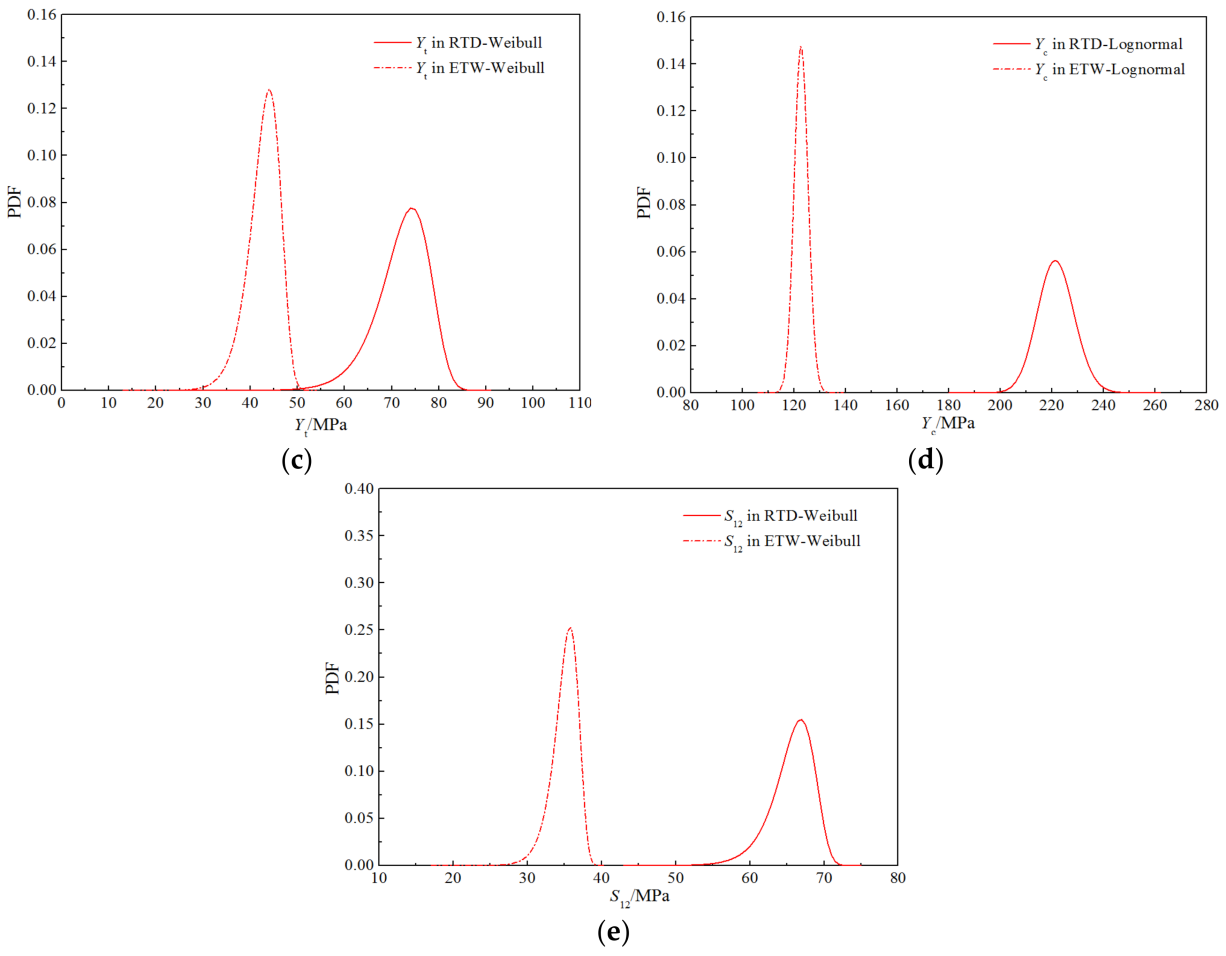
Comparison of the probability curves for the strength properties of H-GFRPs. (**a**) *X*_t_, (**b**) *X*_c,_ (**c**) *Y*_t_, (**d**) *Y*_c,_ (**e**) *S*_12_.

Figure 15 presents the probability density functions for the strength properties of the H-GFRPs under the two working conditions. Some differences are apparent in the peak positions of the curves for longitudinal compressive strength under the two working conditions, but the curve shapes are similar; hence, the humid and hot environment has little impact on the probability distribution characteristics of this parameter. By contrast, the curves of the longitudinal tensile, transverse tensile, transverse compressive, and longitudinal and transverse shear strengths under the two working conditions have different curve shapes and significantly different peak positions; thus, the humid and hot environment has a significant influence on these four parameters.

### 3.2. Determining the Probability Distribution of the Mechanical Properties of Materials Based on Micromechanical Model

#### 3.2.1. Method

The literature shows that the method of analysing the statistical characteristics of the mechanical properties of materials based on the meso-scale is more efficient than that based on test data. The process of determining the probability distribution characteristics of the composite mechanical properties based on the meso-scale method is as follows:

(1) Determine the statistical characteristics of the component material performance parameters.
(2) Conduct a random number of simulations based on the statistical characteristics of the component materials to obtain the random input parameters.
(3) Substitute the random input parameters into the meso-mechanical theoretical model to obtain randomly distributed composite stiffness properties.

It can be seen that the key factors in this method include the statistical characteristics of component materials and the theoretical model of micromechanics.

Although researchers have developed several micromechanical theoretical models, most of them can only predict the stiffness properties of composites. Research shows that among the existing theoretical micromechanical models, the bridging model [30] can predict the stiffness properties of composites more accurately. Therefore, the bridging model was used to calculate the four basic stiffness properties of the composites, which are expressed as:

(2) $$\begin{array}{l} E_{11}=V_{f}E_{f}+V_{m}E_{m}\\ v_{12}=V_{f}v_{f}+V_{m}v_{m}\\ E_{22}=\frac{\left(V_{f}+V_{m}a_{11}\right)\left(V_{f}+V_{m}a_{22}\right)}{\left(V_{f}+V_{m}a_{11}\right)\left(V_{f}S_{22}^{f}+a_{22}V_{m}S_{22}^{m}\right)+V_{f}V_{m}\left(S_{21}^{m}-S_{21}^{f}\right)a_{12}}\\ G_{12}=G_{m}\frac{\left(G_{f}+G_{m}\right)+V_{f}\left(G_{f}-G_{m}\right)}{\left(G_{f}+G_{m}\right)-V_{f}\left(G_{f}-G_{m}\right)} \end{array}$$

where $a_{11}=E_{m}/E_{f}$, $a_{22}=0.3+0.7\left(E_{m}/E_{f}\right)$, $a_{12}=\left(S_{12}^{f}-S_{12}^{m}\right)\left(a_{11}-a_{22}\right)/\left(S_{11}^{f}-S_{11}^{m}\right)$, $S_{11}^{f}=S_{22}^{f}=1/E_{f}$, $S_{12}^{f}=S_{21}^{f}=-\nu_{f}/E_{f}$, $S_{11}^{m}=S_{22}^{m}=1/E_{m}$, and $S_{12}^{m}=S_{21}^{m}=-\nu_{m}/E_{m}$. *E_f_*, *v_f_*, and *G_f_* denote the elastic modulus, Poisson’s ratio, and shear modulus of the glass fibre, respectively, meeting the requirements of $G_{f}=E_{f}/2\left(1+\nu_{f}\right)$; *E_m_*, *v_m_*, and *G_m_* represent the elastic modulus, Poisson’s ratio, and shear modulus of the matrix, respectively, meeting the requirements of $G_{m}=E_{m}/2\left(1+\nu_{m}\right)$; and *V_f_* and *V_m_* denote the volume contents of the fibre and matrix, respectively. When the void content is ignored, they satisfy the requirement of *V_f_* + *V_m_* = 1.

It can be seen from the calculation formula of the above bridging model that the mutually independent variables in the model include *E_f_*, *v_f_*, *E_m_*, *v_m_*, and *v_f_*. When using the model to determine the statistical characteristics of composite stiffness properties, it is necessary to first determine the statistical characteristics of the mutually independent random variables.

#### 3.2.2. Prediction Model of the Stiffness Performance of Component Materials

The normalised temperature T* was used to fit the functional relationship between the mechanical properties of the composites and the hygrothermal environment. The normalised temperature is defined as follows [31]:

(3) $$T^{*}=\frac{T_{g}-T}{T_{g}^{0}-T_{0}}$$

where *T*_g_ and $T_{g}^{0}$ denote the glass transition temperatures of the composites in the hygroscopic and dry states, respectively; *T* and *T*_0_ denote the test and standard laboratory temperatures, respectively; and $T_{g}^{0}$ denotes the glass transition temperature of the composite in the hygroscopic state, which can be calculated from [31].

(4) $$T_{g}=T_{g}^{0}-kC$$

where *K* denotes the temperature offset in unit hygroscopicity and *C* denotes the moisture absorption. The glass transition temperature of the composites decreased with an increase in the hygroscopic capacity. The moisture absorption of the composite material was calculated using the following formula:

(5) $$C=\frac{m-m_{0}}{m_{0}}\times 100\%$$

where *m* denotes the mass of the test piece after hygroscopicity and *m*_0_ denotes the mass of the test piece in the dry state.

The properties of the H-GFRP components in the RTD condition are listed in Table 13. The volume fraction of the glass fibre was 60%, and that of the epoxy resin matrix was 40% when the void volume content was ignored. Glass fibre and epoxy resin matrix are isotropic materials, corresponding to two independent elastic parameters. The shear modulus can be obtained from the elastic modulus and Poisson’s ratio.

The method based on the meso-mechanical model uses the meso-scale, and the material properties (fibre and matrix performance parameters, fibre volume content, etc.) of the composite materials in humid and hot environments were combined with a meso-mechanical theoretical model to determine the material performance parameters under humid and hot environment conditions. As the bridging model can accurately predict the mechanical properties of H-GFRPs, the component material parameter model considering the damp-heat effect is described by:

(6) $$\begin{array}{l} \frac{E_{f}^{hyg}}{E_{f}^{0}}=\frac{G_{f}^{hyg}}{G_{f}^{0}}={\left(T^{*}\right)}^{i},\;\frac{\nu_{f}^{hyg}}{\nu_{f}^{0}}=1\\ \frac{E_{m}^{hyg}}{E_{m}^{0}}=\frac{G_{m}^{hyg}}{G_{m}^{0}}={\left(T^{*}\right)}^{j},\;\frac{\nu_{m}^{hyg}}{\nu_{m}^{0}}=1 \end{array}$$

where *E_f_*, *v_f_*, and *G_f_* denote the elastic modulus, Poisson’s ratio, and shear modulus of the fibre, respectively; and *E_m_*, *v_m_*, and *G_m_* denote the elastic modulus, Poisson’s ratio, and shear modulus of the matrix, respectively. The superscripts “hyg” and “0” represent ETW and RTD, respectively. Exponential parameters *i* and *j* are material parameters obtained via fitting. On conducting an extensive literature review, it was found that the mechanical properties of fibres are less affected by the humid and hot environment and so the exponential parameter *i* = 0.04, whereas the mechanical properties of the matrix are more affected by the humid and hot environment, and so the exponential parameter *j* = 0.5. The normalized temperature T* can be determined from Equation (3). According to Equation (6), component material parameters under ETW can be obtained.

#### 3.2.3. Probability Distribution Analysis of the Stiffness Properties of Materials in the RTD State

**Figure 16:**
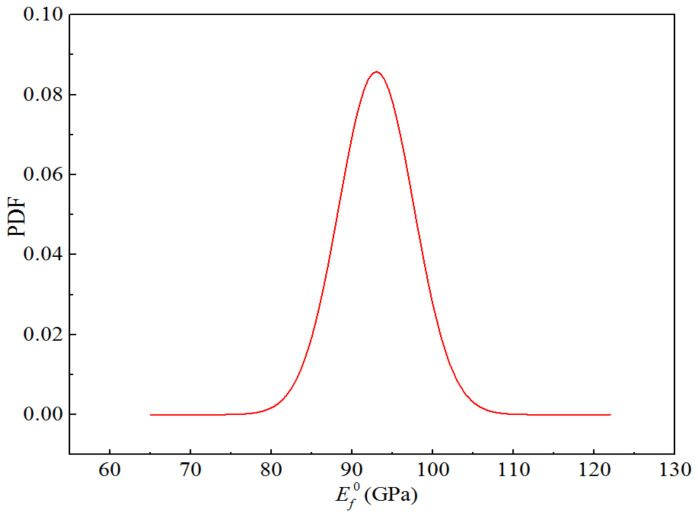
Probability function diagram for the elastic modulus of H-GFRP in the RTD state.

The fibre volume content of H-GFRPs was 60%. Glass fibre and epoxy resin matrix are isotropic materials associated with two independent elastic parameters. The performance parameters of the component materials are listed in Table 14. The CV of all random parameters was 0.05 [20,21], and the distribution type was a normal distribution. The five random variables were independent of each other. Considering the elastic modulus of the glass fibre as an example, the probability density function for the random variables is presented in Figure 16.

**Figure 17:**
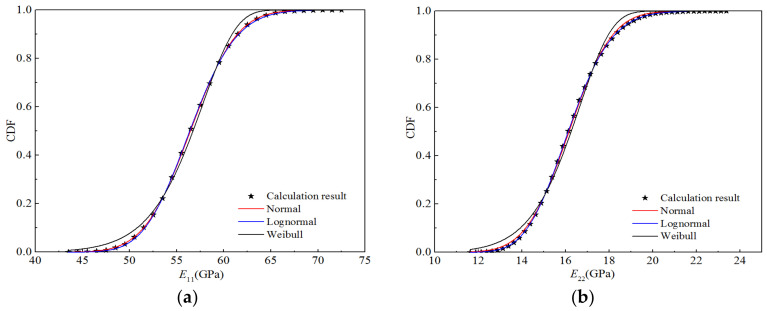

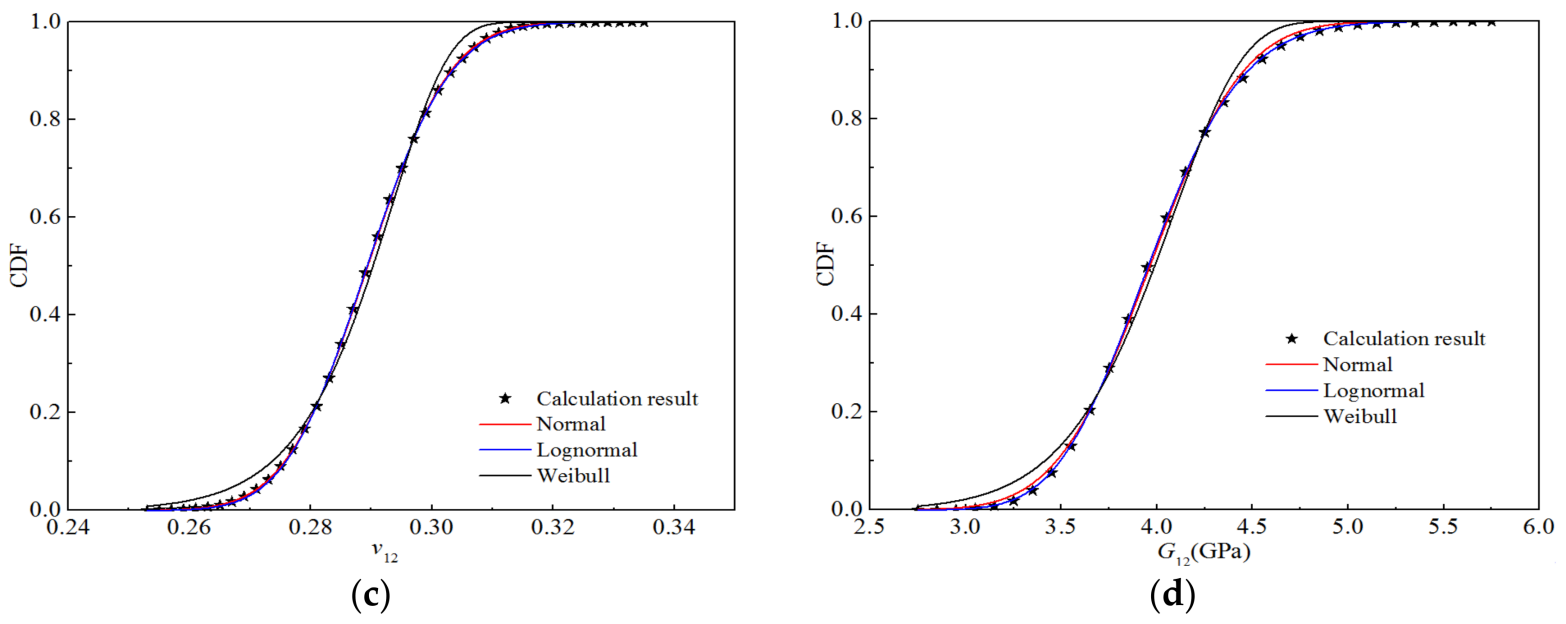
Function fitting curves for the stiffness of H-GFRPs under the RTD condition. (**a**) *E*_11_, (**b**) *E*_22,_ (**c**) *v*_12_, (**d**) *G*_12_.

According to the distribution parameters for the random variables presented in Table 14, a random number was generated in MATLAB^®^, and the number of samples was 10,000; hence, 10,000 sample points for stiffness performance were calculated using the bridging model. Using Origin^®^ for the four stiffness properties, the appropriate interval was selected for frequency statistics to obtain the corresponding cumulative frequency and cumulative probability distribution for each elastic parameter, as shown in Figure 17. Using Origin^®^ with the normal distribution, the CDFs of the four elastic parameters were obtained through fitting the lognormal and Weibull distributions. Figure 17 also displays the fitting curves for the three distribution types. It can be seen that the CDFs fitted by the normal and lognormal distributions are basically consistent with the calculated data points, whereas that fitted by the Weibull distribution exhibits certain deviations from the calculated values, particularly at the upper and lower tails of the curve.

Table 15 shows the distribution parameters and goodness-of-fit values corresponding to the three distribution fitting curves. As can be seen, the longitudinal tensile–compression modulus *E*_11_ and Poisson’s ratio *v*_12_ tend to follow a normal distribution, whereas the transverse tensile–compression modulus *E_22_* and longitudinal and transverse shear modulus *G*_12_ tend to follow a lognormal distribution. The goodness-of-fit of the best distribution was greater than 0.99998.

**Figure 18:**
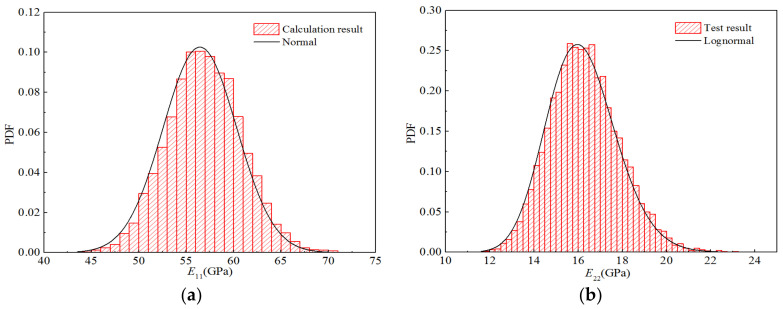

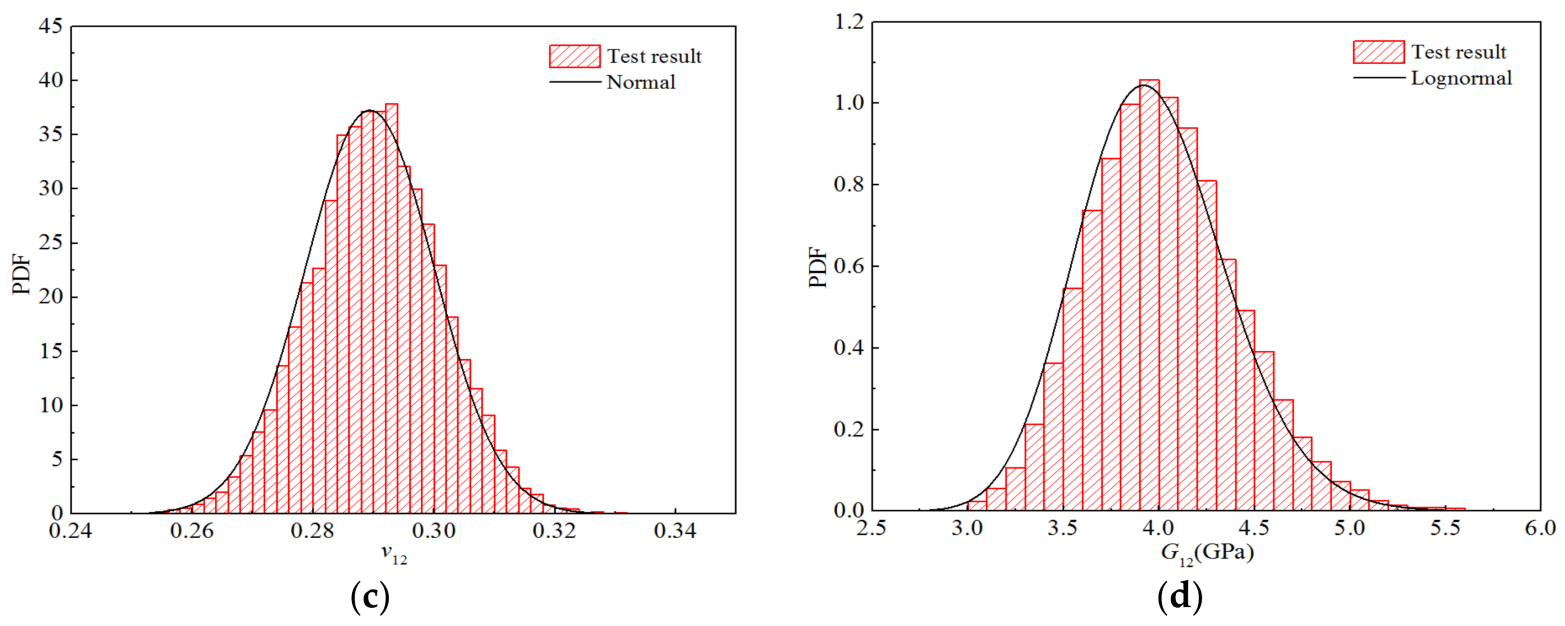
Probability distribution diagrams for the stiffness properties of H-GFRPs under RTD conditions. (**a**) *E*_11_, (**b**) *E*_22,_ (**c**) *v*_12_, (**d**) *G*_12_.

Furthermore, according to the frequency statistics listed above, the corresponding frequencies can be obtained to calculate the probability density distribution histogram for each elastic parameter, as displayed in Figure 18. The probability density function of the optimal distribution is also shown in the figure. It can be seen that the curves for the optimal distributions are in good agreement with the obtained histograms.

**Figure 19:**
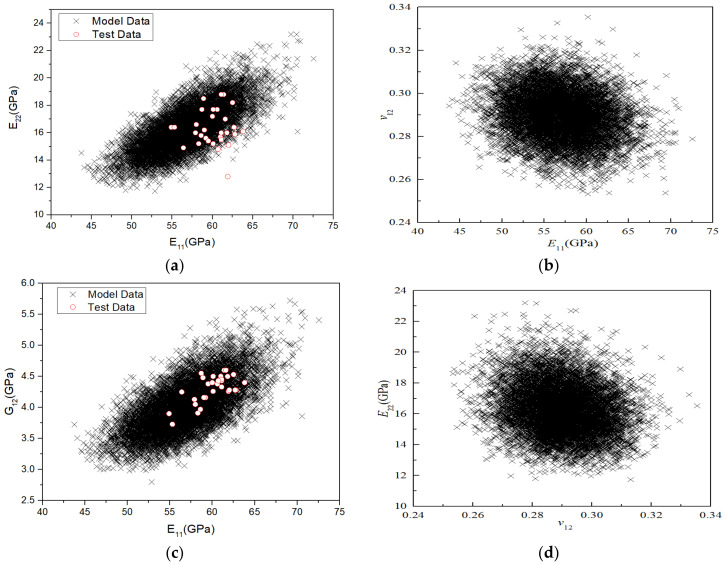

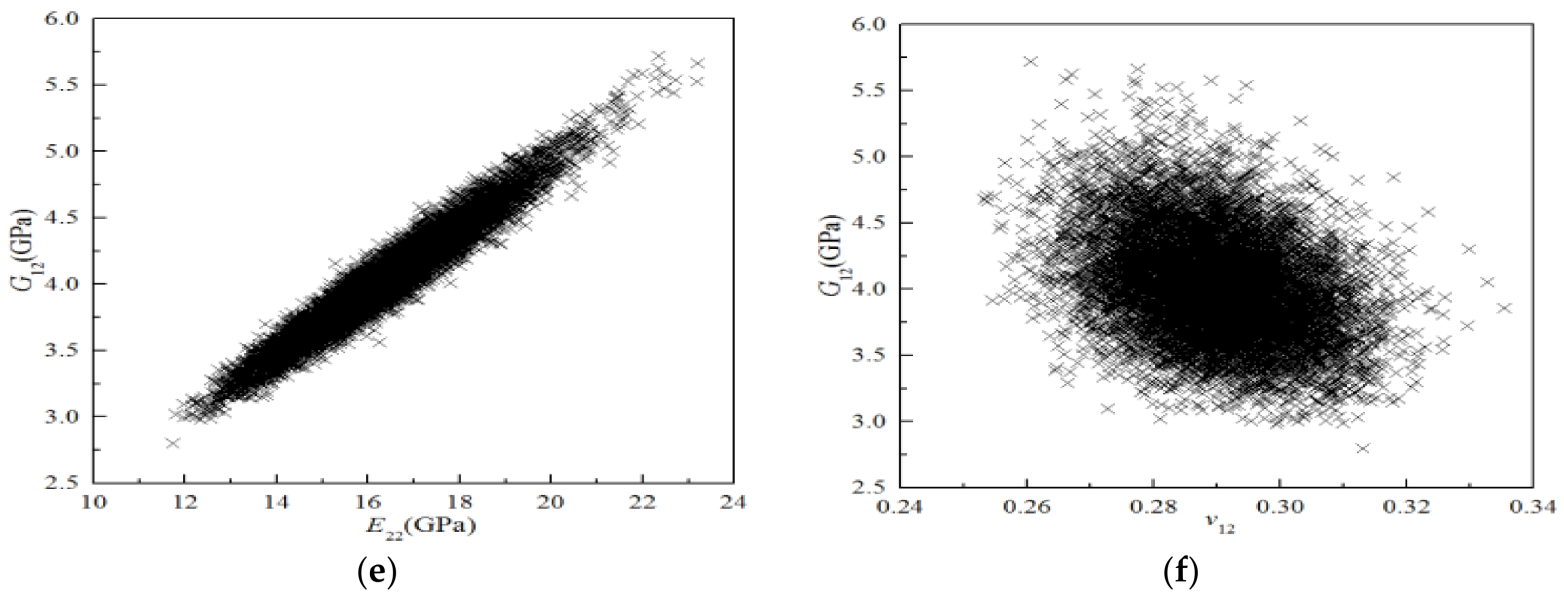
Correlations between the stiffness properties of H-GFRPs under the RTD condition. (**a**) *E*_11_ and *E*_22_, (**b**) *E*_11_ and *v*_12,_ (**c**) *E*_11_ and *G*_12_, (**d**) *E*_22_ and *v*_12,_ (**e**) *E*_22_ and *G*_12_, (**f**) *v*_12_ and *G*_12_.

Furthermore, according to the micromechanical analysis, the correlation between various stiffness properties can be obtained. To qualitatively analyse the correlation between the stiffness properties, 10,000 sampling points were drawn in the coordinate system to obtain the correlation diagram between various stiffness properties, as shown in Figure 19. The closer the shape of the data point distribution is to a circle, the weaker the correlation; the closer it is to an ellipse and the higher the ratio of the long axis to the short axis of the ellipse, the stronger the correlation. As Figure 19 shows, the correlation between *v*_12_ and the other three stiffness parameters is weak, whereas that between the other three stiffness parameters is strong. Figure 19a,c show that the correlation numerical data between *E*_11_*–E*_22_ and *E*_11_*–G*_12_ completely covered the test results, indicating that the model can well predict the correlation of stiffness performance under RTD.

Furthermore, the Pearson correlation coefficient was used to quantitatively describe the correlation between stiffness and performance. Equation (7) presents the calculation formula for Pearson’s correlation coefficient. The correlation coefficients are in the range of [−1.0, 1.0]; values close to 0 indicate that the two variables are uncorrelated or weakly correlated, whereas values close to −1.0 or 1.0 indicate stronger correlation. Correlation coefficient values in the range of (0, 1) represent positive correlations, whereas those in the range of [−1, 0) represent negative correlations.

(7) $$\rho \left(X,Y\right)=\frac{\sum XY-\frac{\sum X\sum Y}{N}}{\sqrt{\left(\sum X^{2}-\frac{{\left(\sum X\right)}^{2}}{N}\right)\left(\sum Y^{2}-\frac{{\left(\sum Y\right)}^{2}}{N}\right)}}$$

Here, *x* and *y* are random variables and *N* is the number of sample variables. The correlation between the parameters can be calculated from 10,000 sampling points of the material stiffness performance, as shown in Table 16. It can be seen from Table 16 that *E*_11_ is positively correlated with *E*_22_ and *G*_12_, with a correlation coefficient of approximately 0.7, and weakly correlated with *v*_12_ (−0.196); *E*_22_ is positively correlated with *G*_12_ with a correlation coefficient of 0.97, and weakly correlated with *v*_12_, whereas *v*_12_ is negatively correlated with *G*_12_.

#### 3.2.4. Probability Distribution Analysis of Material Stiffness Performance in ETW

Among the four humid and hot working conditions tested, the worst working condition was the combination of a high temperature of 70 °C and balanced moisture absorption. Therefore, the probability distribution characteristics of the mechanical properties of the material in this case are analysed in Section 3.2.1.

**Figure 20:**
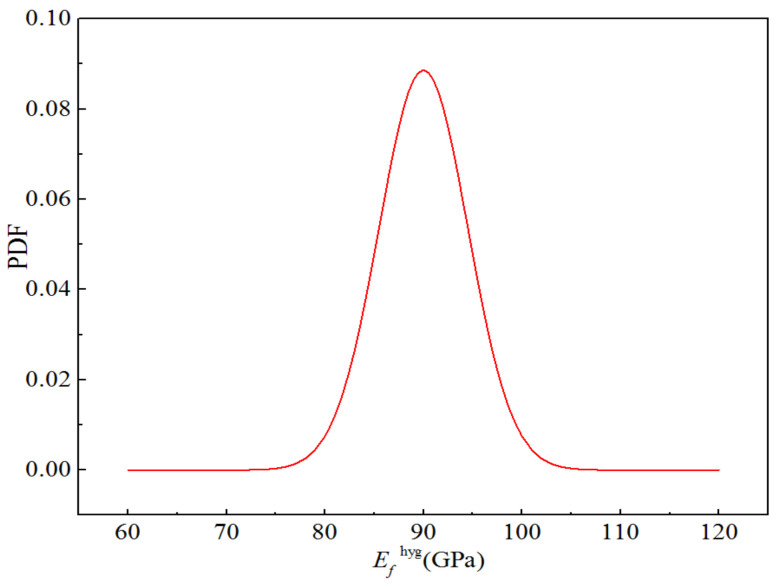
Probability distribution for the elastic modulus of H-GFRPs under the ETW condition.

Table 17 lists the performance parameters of the component materials in a humid and hot environment. The glass fibre and epoxy resin matrix in H-GFRPs are isotropic materials, and their elastic and shear moduli are affected by humid and hot environment conditions. However, the influence of this environment on the corresponding Poisson’s ratio can be ignored. The elastic and shear moduli of the component materials listed in Table 17 can be calculated using Equation (6). In addition, the influence of humid and hot environment conditions on the fibre volume content was ignored. The CV of all random parameters was 0.05, and the distribution type was normal. The five random parameters were independent of each other. Figure 20 displays the probability density function for the elastic modulus of the glass fibre in the humid and hot environment.

**Figure 21:**
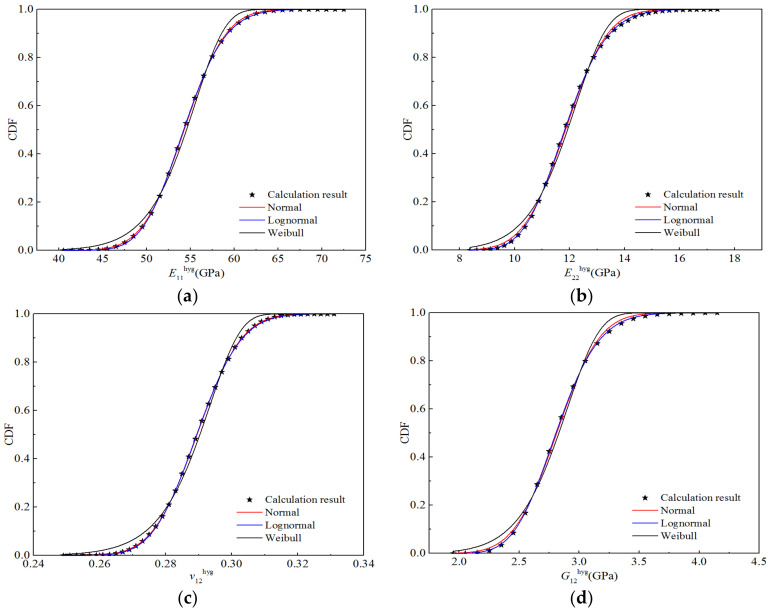
Function fitting curves for the stiffness of H-GFRPs under the ETW condition. (**a**) *E*_11_, (**b**) *E*_22,_ (**c**) *v*_12_, (**d**) *G*_12_.

According to the distribution parameters for the random variables listed in Table 16, a random number with a sample size of 10,000 was generated in MATLAB^®^, and 10,000 sample points for the stiffness properties of H-GFRPs under a humid and hot environment were calculated using the bridging model. In Origin^®^, frequency statistics were created based on the stiffness performance sample points to obtain the corresponding cumulative frequency and cumulative probability distribution relative to each performance parameter, as shown in Figure 21. Additionally, the CDFs for the four stiffness performance parameters were obtained through fitting with the lognormal and Weibull distributions. It is apparent that the CDFs fitted by the normal and lognormal distributions are basically consistent with the calculated data points, whereas that fitted by the Weibull distribution exhibits certain deviation from the calculated value, particularly at the upper and lower tails of the curve.

Table 18 presents the distribution parameters and goodness-of-fit results corresponding to the three distribution fitting curves. It can be seen that the longitudinal tensile–compression modulus *E*_11_, transverse tensile–compression modulus *E*_22_, and longitudinal and transverse shear modulus *G*_12_ tend to obey a lognormal distribution, whereas Poisson’s ratio *v*_12_ tends to obey a normal distribution. The goodness-of-fit values for the best distributions of the four stiffness performance parameters were ≥0.99995.

**Figure 22:**
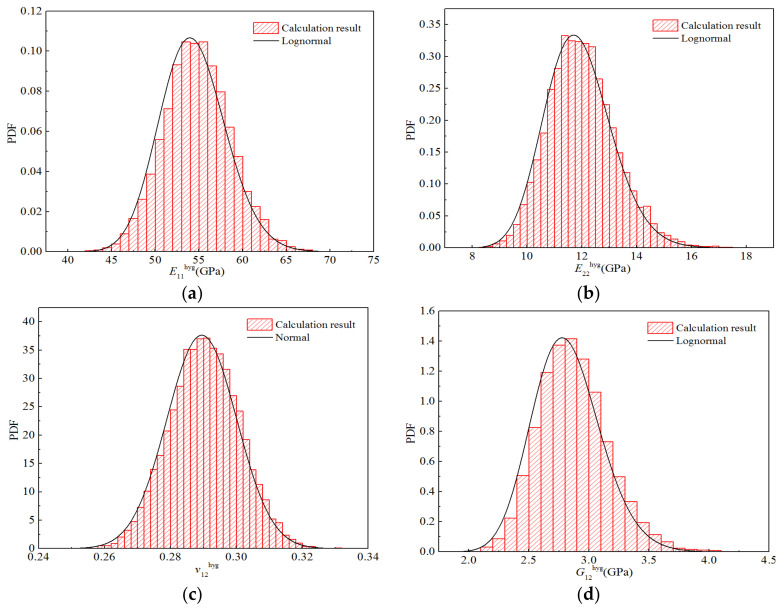
Probability distribution diagrams for the stiffness properties of H-GFRPs under the ETW condition. (**a**) *E*_11_, (**b**) *E*_22,_ (**c**) *v*_12_, (**d**) *G*_12_.

Moreover, according to the frequency statistics listed above, the corresponding frequency can be obtained to calculate the probability density distribution histogram for each stiffness performance parameter, as shown in Figure 22. Figure 11 also shows the probability density function of the optimal distribution. It can be observed that the optimal distribution curves of the four stiffness performance parameters are in good agreement with the obtained histograms.

**Figure 23:**
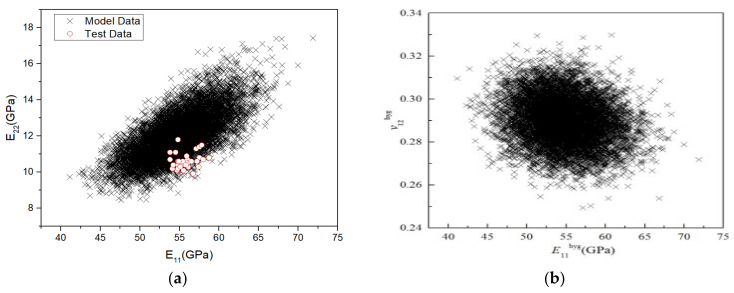

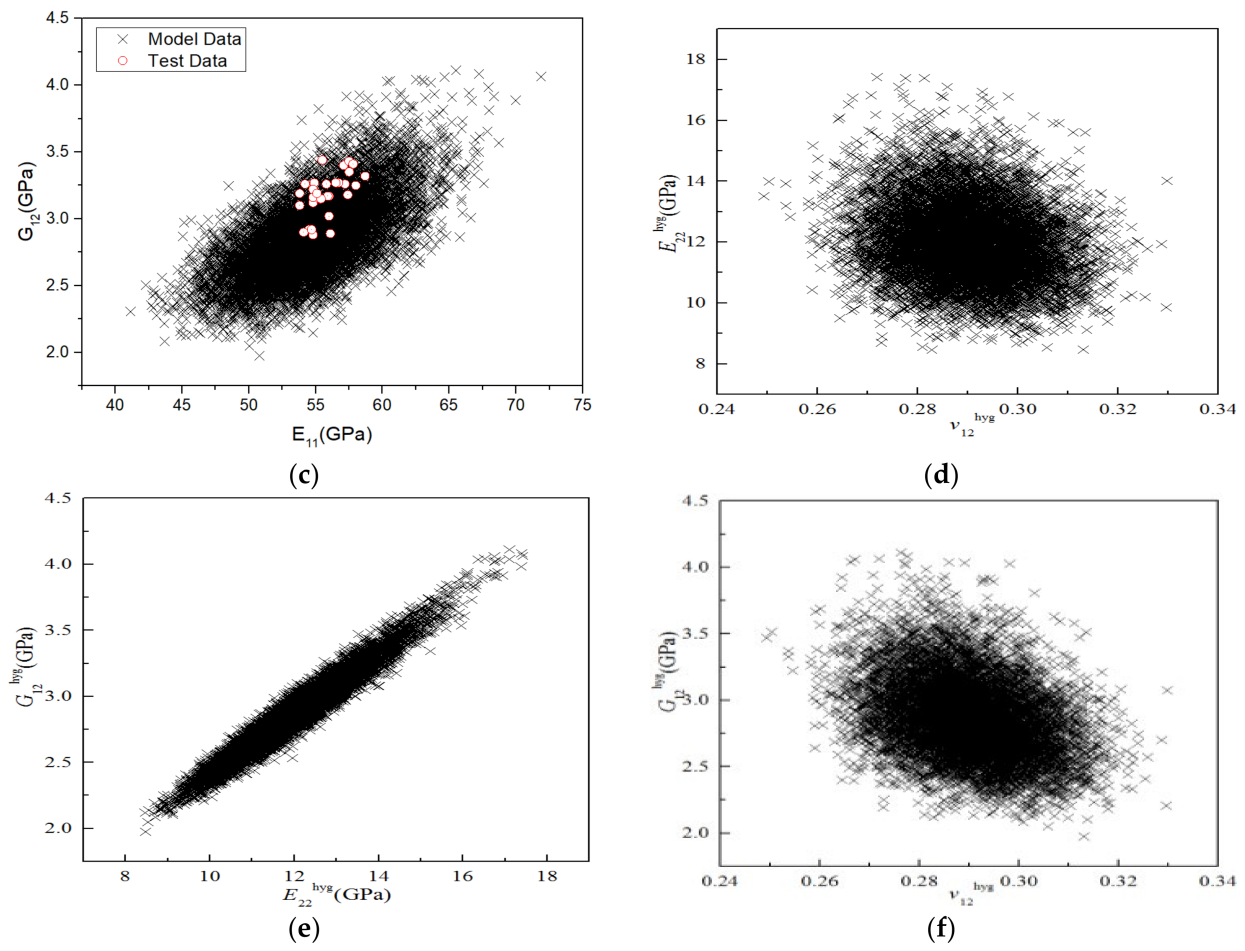
Diagrams of correlation between the stiffness properties of H-GFRPs under the ETW condition. (**a**) *E*_11_ and *E*_22_, (**b**) *E*_11_ and *v*_12,_ (**c**) *E*_11_ and *G*_12_, (**d**) *E*_22_ and *v*_12,_ (**e**) *E*_22_ and *G*_12_, (**f**) *v*_12_ and *G*_12_.

Furthermore, the correlation between various stiffness performance parameters was obtained through a micromechanical analysis. To qualitatively analyse the correlation between the stiffness properties, 10,000 sampling points were drawn in the coordinate system to obtain the correlation diagram between various stiffness properties, as shown in Figure 23. The more circular the shape of the data point distribution, the weaker the correlation; the closer the shape is to an ellipse and the higher the ratio of the long axis to the short axis of the ellipse, the stronger the correlation. It can be seen that the correlation between *v*_12_ and the other three stiffness parameters is weak, whereas that between the other three stiffness parameters is strong. Figure 23a,c show that the correlation numerical data between *E*_11_–*E*_22_ and *E*_11_–*G*_12_ completely covered the test results, indicating that the model can well predict the correlation of stiffness performance under ETW.

Moreover, the Pearson linear correlation coefficient between the parameters could be calculated according to the 10,000 sampling points of the material stiffness performance; the results are displayed in Table 19. It is clear that *E*_11_ is positively correlated with *E*_22_ and *G*_12_, with a correlation coefficient of approximately 0.7, and weakly correlated with *v*_12_ (−0.196). *E*_22_ is positively correlated with *G*_12_, with a correlation coefficient of 0.97, and weakly correlated with *v*_12_, whereas *v*_12_ is negatively correlated with *G*_12_.

#### 3.2.5. Comparison of the Probability Distribution Characteristics of Stiffness Performances under the RTD and Hot and Humid Environment Conditions

To analyse the influence of humid and hot environments on the statistical characteristics of the stiffness properties of H-GFRPs, the probability distribution characteristics of the stiffness properties in the RTD state and humid and hot environment determined in Section 3.2.2 and Section 3.2.3 were considered.

Table 20 presents the RTD and humid environment details, probability distribution types, and distribution parameters corresponding to stiffness performance under 70 °C high temperature and equilibrium moisture absorption. It can be seen that although the longitudinal tensile–compression modulus *E*_11_ obeys the normal distribution in the RTD state and the lognormal distribution under the hot and humid environment, the distribution types of the other parameters under the two working conditions are similar; more precisely, the transverse tensile–compression modulus *E*_22_ and the shear modulus *G*_12_ obey the lognormal distribution under both working conditions. It should be noted that Poisson’s ratio *v*_12_ exhibits similar distributions under the two working conditions because the influence of the humid and hot environment on Poisson’s ratio and the volume fraction of the component materials was neglected. As mentioned above, the lognormal and normal distributions are equivalent in describing the statistical characteristics of the mechanical property parameters of materials. It can be observed that a humid and hot environment does not alter the probability distribution type of the stiffness properties of H-GFRPs.

**Figure 24:**
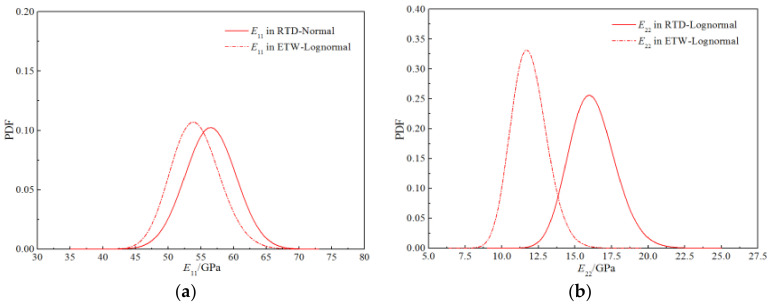

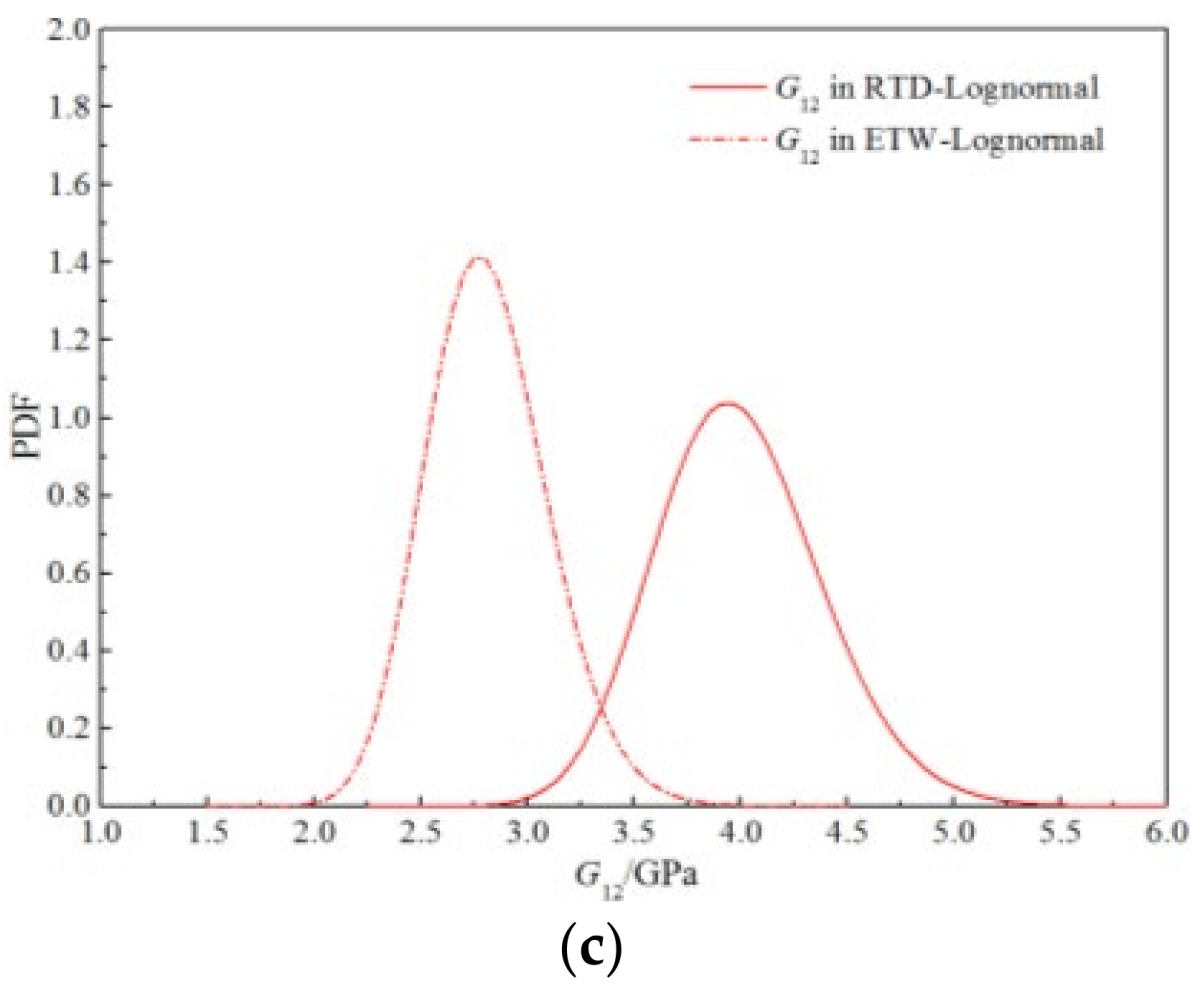
Comparison of the probability distribution curves for stiffness properties of H-GFRPs under different conditions. (**a**) *E*_11_, (**b**) *E*_22,_ (**c**) *G*_12_.

Figure 24 exhibits the probability density functions of the stiffness performances of the H-GFRPs under the two working conditions. It can be seen that the peak positions of the curves for the longitudinal tensile–compression modulus under the two working conditions are relatively close, and the curve shapes are similar; thus, the humid and hot environment has little impact on the probability distribution characteristics of this parameter. The peak positions of the curves for the transverse tensile–compression modulus and longitudinal and transverse shear modulus under the two working conditions are quite different, exhibiting a certain difference in the width; thus, the humid and hot environment has a significant impact on these two parameters.

## 4. Conclusions

In this study, the dispersion of the mechanical properties of different batches of H-GFRPs was analysed based on test data. A probability distribution analysis method for the mechanical properties based on the macroscopic test data and a meso-mechanical model was proposed. The following conclusions were drawn:

(1) The mechanical properties of different batches did not become unstable in hot and humid environments; however, with the deterioration of hot and humid conditions, the dispersion of performance tended to decrease.
(2) The probability distribution characteristics of the stiffness and strength performances were obtained using the macroscopic test data. Under hot and humid conditions, the distribution types of other parameters are consistent with those under RT conditions, except for the transverse tensile modulus *E*_22,t_ and longitudinal compressive strength *X*_c_, which tend to follow a normal distribution.
(3) The probability distributions of the stiffness performances and their correlations were obtained using a method based on the meso-mechanical model. Among the four stiffness performance parameters, the correlation between *v*_12_ and the other three stiffness parameters was weak, whereas that between the other three stiffness parameters was strong.

## Figures and Tables

**Table 1:** Working conditions considered in the mechanical property test.

No.	Temperature	Moisture Absorption	Code
I	−55 °C	dry state	CTD
II	23 °C	dry state	RTD
III	80 °C	dry state	ETD
IV	55 °C	equilibrium moisture absorption	ETW1
V	70 °C	equilibrium moisture absorption	ETW

Note: condition II is standard laboratory environment.

**Table 2:** Tests used to determine the basic mechanical properties.

Type of Test	Specimen Size (Length × Width × Thickness)	Stiffness Properties	Strength Properties
Tensile test (0°)	250 mm × 15 mm × 1 mm	Longitudinal tensile modulus *E*_11,t_, Poisson’s ratio *v*_12_	Longitudinal tensile strength *X*_t_
Compression test (0°)	140 mm × 12 mm × 2 mm	Longitudinal compression modulus *E*_11,c_	Longitudinal compressive strength *X*_c_
Tensile test (90°)	175 mm × 25 mm × 2 mm	Transverse tensile modulus *E*_22,t_	Transverse tensile strength *Y*_t_
Compression test (90°)	140 mm × 12 mm × 2 mm	Transverse compression modulus *E*_22,c_	Transverse compressive strength *Y*_c_
In-plane shear test	250 mm × 25 mm × 3.6 mm	Longitudinal and transverse shear modulus *G*_12_	Longitudinal and transverse shear strength *G*_12_

**Table 3:** Batches of high-strength glass fibre composites (H-GFRPs).

Batch	Date
1	4 November 2017
2	27 November 2017
3	29 November 2017
4	30 November 2017
5	22 January 2018

**Table 4:** Statistical values of stiffness properties of five batches of H-GFRP.

**No.**	***E*_11,t_/GPa**			***E*_11,c_/GPa**			***E*_22,t_/GPa**		
	**Mean**	**SD**	**CV/%**	**Mean**	**SD**	**CV/%**	**Mean**	**SD**	**CV/%**
I	58.5	2.71	4.63	61.0	1.32	2.17	17.7	1.29	7.29
II	60.0	1.72	2.87	61.1	0.51	0.84	16.3	1.26	7.72
III	55.4	0.29	0.53	59.6	1.10	1.85	13.5	0.82	6.06
IV	57.1	1.75	3.07	59.4	0.58	0.97	10.6	0.25	2.36
V	56.3	2.34	4.15	58.4	0.71	1.21	8.90	0.19	2.19
**No.**	***E*_22,c_/GPa**			***G*_12_/GPa**			***v*_12_**		
	**Mean**	**SD**	**CV/%**	**Mean**	**SD**	**CV/%**	**Mean**	**SD**	**CV/%**
I	19.8	0.76	3.83	5.43	0.357	6.58	/	/	/
II	17.7	0.88	4.96	4.30	0.211	4.90	0.283	0.0067	2.36
III	15.3	0.76	4.93	3.51	0.152	4.32	/	/	/
IV	14.9	0.43	2.91	3.18	0.172	5.40	/	/	/
V	13.4	0.25	1.86	2.76	0.074	2.69	/	/	/

**Table 5:** Statistical values of strength properties of five batches of H-GFRP.

**No.**	***X*_t_/MPa**			***X*_c_/MPa**			***Y*_t_/MPa**		
	**Mean**	**SD**	**CV/%**	**Mean**	**SD**	**CV/%**	**Mean**	**SD**	**CV/%**
I	2151	144	6.71	1392	83	5.95	91.8	6.45	7.02
II	1961	103	5.26	1216	55	4.54	73.3	3.06	4.17
III	1683	74	4.38	1014	108	10.60	61.5	2.57	4.18
IV	1102	40	3.66	975	77	7.85	51.9	1.67	3.22
V	1009	66	6.49	859	62	7.21	43.5	1.72	3.97
**NO.**	***Y*_c_/MPa**			***S*_12_/MPa**			**/**	**/**	**/**
	**Mean**	**SD**	**CV/%**	**Mean**	**SD**	**CV/%**	**/**	**/**	**/**
I	310	15.01	4.85	88.0	3.21	3.65	/	/	/
II	223	5.96	2.67	65.8	1.50	2.27	/	/	/
III	164	5.61	3.42	50.1	1.52	3.03	/	/	/
IV	141	4.04	2.86	42.0	2.52	6.00	/	/	/
V	123	1.30	1.06	35.2	1.05	2.99	/	/	/

**Table 6:** Statistical values of the longitudinal and transverse tensile−compression moduli of the five H-GFRP batches.

No.	*E*_11_/GPa			*E*_22_/GPa		
	Mean	SD	CV/%	Mean	SD	CV/%
I	59.8	1.25	2.09	18.76	0.535	2.85
II	60.5	0.95	1.56	17.03	0.845	4.96
III	57.5	0.59	1.02	14.43	0.478	3.31
IV	58.3	1.07	1.83	12.76	0.182	1.42
V	57.4	1.28	2.23	11.15	0.150	1.35

**Table 7:** Statistical distribution parameters for the stiffness properties of H-GFRPs at RT.

Model	Normal			Lognormal			Weibull		
	*μ*	*σ*	*Adj. R* ^2^	*μ*	*σ*	*Adj. R* ^2^	*λ*	*κ*	*Adj. R* ^2^
*E*_11,t_/GPa	59.50	2.40	0.996	4.09	0.040	0.996	60.43	29.24	0.992
*E*_11,c_/GPa	60.92	1.39	0.980	4.11	0.023	0.979	61.47	50.50	0.986
*E*_22,t_/GPa	16.09	1.28	0.983	2.78	0.079	0.986	16.59	14.22	0.971
*E*_22,c_/GPa	17.49	1.07	0.997	2.86	0.061	0.997	17.92	18.75	0.989
*v*_12_	0.281	0.015	0.995	−1.27	0.053	0.994	0.286	22.44	0.993
*G*_12_/GPa	4.28	0.229	0.988	1.45	0.053	0.985	4.37	21.95	0.995

**Table 8:** Values of the three statistical distribution parameters for the strength properties of H-GFRPs at RT.

Model	Normal			Lognormal			Weibull		
	*μ*	*σ*	*R*	*μ*	*σ*	*R*	*λ*	*κ*	*R*
*X*_t_/MPa	1926	171	0.993	7.56	0.088	0.990	1992	13.1	0.997
*X*_c_/MPa	1183	89	0.994	7.08	0.076	0.996	1217	15.5	0.985
*Y*_t_/MPa	72.53	5.45	0.989	4.28	0.075	0.986	74.62	15.75	0.997
*Y*_c_/MPa	222	7.15	0.986	5.40	0.032	0.987	225	35.40	0.967
*S*_12_/MPa	65.90	2.87	0.930	4.19	0.043	0.922	66.95	28.21	0.955

**Table 9:** Values of the three statistical distribution parameters for the stiffness properties of H-GFRPs in a humid and hot environment.

Model	Normal			Lognormal			Weibull		
	*μ*	*σ*	*Adj. R* ^2^	*μ*	*σ*	*Adj. R* ^2^	*λ*	*κ*	*Adj. R* ^2^
*E*_11,t_/GPa	55.48	2.88	0.990	4.02	0.052	0.991	56.64	21.70	0.977
*E*_11,c_/GPa	58.30	1.13	0.995	4.07	0.019	0.994	58.75	59.61	0.995
*E*_22,t_/GPa	8.83	0.38	0.976	2.18	0.043	0.976	8.98	26.36	0.971
*E*_22,c_/GPa	13.33	0.45	0.992	2.59	0.034	0.992	13.51	34.19	0.986
*G*_12_/GPa	2.76	0.097	0.971	1.01	0.035	0.969	2.79	33.18	0.988

**Table 10:** Values of the three statistical distribution parameters for the strength properties of H-GFRPs under the ETW condition.

Model	Normal			Lognormal			Weibull		
	*μ*	*σ*	*Adj. R* ^2^	*μ*	*σ*	*R*	*λ*	*κ*	*Adj. R* ^2^
*X*_t_/MPa	988	93	0.993	6.89	0.094	0.990	1024	12.3	0.997
*X*_c_/MPa	829	99	0.998	6.72	0.119	0.998	867	9.7	0.996
*Y*_t_/MPa	42.99	3.26	0.986	3.76	0.076	0.984	44.24	15.40	0.987
*Y*_c_/MPa	122	2.71	0.995	4.81	0.022	0.996	124	52.19	0.985
*S*_12_/MPa	35.19	1.62	0.980	3.56	0.045	0.978	35.80	24.63	0.989

**Table 11:** Comparison of the probability distribution characteristics of the stiffness properties of H-GFRPs.

Stiffness Property	RTD			ETW		
	*μ* (*λ*)	*σ* (*κ*)	Distribution	*μ* (*λ*)	*σ* (*κ*)	Distribution
*E*_11,t_/GPa	4.09	0.040	Lognormal	4.02	0.052	Lognormal
*E*_11,c_/GPa	61.47	50.50	Weibull	58.75	59.61	Weibull
*E*_22,t_/GPa	2.78	0.079	Lognormal	8.83	0.38	Normal
*E*_22,c_/GPa	2.86	0.061	Lognormal	2.59	0.034	Lognormal
*v*_12_	0.281	0.015	Normal	/	/	/
*G*_12_/GPa	4.37	21.95	Weibull	2.79	33.18	Weibull

**Table 12:** Comparison of the probability distributions of the strength properties of H-GFRPs.

Stiffness Property	RTD			ETW		
	*μ* (*λ*)	*σ* (*κ*)	Distribution	*μ* (*λ*)	*σ* (*κ*)	Distribution
*X*_t_/MPa	1992	13.1	Weibull	1024	12.3	Weibull
*X*_c_/MPa	7.08	0.076	Lognormal	829	99	Normal
*Y*_t_/MPa	74.62	15.75	Weibull	44.24	15.40	Weibull
*Y*_c_/MPa	5.40	0.032	Lognormal	4.81	0.022	Lognormal
*S*_12_/MPa	66.95	28.21	Weibull	35.80	24.63	Weibull

**Table 13:** Performance parameters of H-GFRPs components under the RT condition.

Volume		Elastic Parameter of Fibre			Elastic Parameter of Matrix		
$V_{f}$	$V_{m}$	$E_{f}$/GPa	$\nu_{f}$	$G_{f}$/GPa	$E_{m}$/GPa	$\nu_{m}$	$G_{m}$/GPa
0.6	0.4	93	0.25	37.2	3.0	0.35	1.11

**Table 14:** Statistical model of the material properties in the RTD state.

Parameter	Mean	CV	Distribution Type
$E_{f}^{0}$/GPa	93	0.05	Normal
$\nu_{f}^{0}$	0.25	0.05	Normal
$E_{m}^{0}$/GPa	3.0	0.05	Normal
$v_{m}^{0}$	0.35	0.05	Normal
$\nu_{f}^{0}$	0.6	0.05	Normal

**Table 15:** Values of the three statistical distribution parameters for the stiffness properties of H-GFRPs under the RTD condition.

Model	Normal			Lognormal			Weibull		
	*μ*	*σ*	*Adj. R* ^2^	*μ*	*σ*	*Adj. R* ^2^	*λ*	*κ*	*Adj. R* ^2^
*E*_11_/GPa	56.45	3.89	0.99998	4.03	0.069	0.99992	57.96	17.01	0.99765
*E*_22_/GPa	16.15	1.56	0.99966	2.78	0.097	0.99999	16.75	12.1	0.99649
*v*_12_	0.289	0.011	0.99999	−1.24	0.037	0.99996	0.294	31.8	0.99743
*G*_12_/GPa	3.96	0.384	0.99961	1.38	0.097	0.99998	4.11	12.05	0.99651

**Table 16:** Correlation coefficients between the stiffness properties of H-GFRPs under the RTD condition.

Correlation Coefficient	*E*_11_	*E*_22_	*v*_12_	*G*_12_
*E*_11_	1	/	/	/
*E*_22_	0.717	1	/	/
*v*_12_	−0.196	−0.189	1	/
*G*_12_	0.662	0.970	−0.338	1

**Table 17:** Statistical model parameters of materials under the ETW condition.

Parameter	*μ*	CV	Model
$E_{f}^{\mathrm{hyg}}$/GPa	90	0.05	Normal
$\nu_{f}^{\mathrm{hyg}}$	0.25	0.05	Normal
$E_{m}^{\mathrm{hyg}}$/GPa	2.08	0.05	Normal
$v_{m}^{\mathrm{hyg}}$	0.35	0.05	Normal
$V_{f}^{\mathrm{hyg}}$	0.6	0.05	Normal

**Table 18:** Values of the three statistical distribution parameters for the stiffness properties of H-GFRPs under the ETW condition.

Model	Normal			Lognormal			Weibull		
	*μ*	*σ*	*Adj. R* ^2^	*μ*	*σ*	*Adj. R* ^2^	*λ*	*κ*	*Adj. R* ^2^
*E*_11_/GPa	54.28	3.75	0.99996	3.99	0.069	0.99997	55.73	16.95	0.99775
*E*_22_/GPa	11.85	1.20	0.99945	2.47	0.102	0.99995	12.31	11.50	0.99602
*v*_12_	0.289	0.011	0.99999	−1.24	0.037	0.99996	0.294	32.18	0.99753
*G*_12_/GPa	2.81	0.282	0.99946	1.03	0.101	0.99995	2.92	11.64	0.99618

**Table 19:** Correlation coefficients between the stiffness properties of H-GFRPs under the ETW condition.

Correlation Coefficient	*E*_11_	*E*_22_	*v*_12_	*G*_12_
*E*_11_	1	/	/	/
*E*_22_	0.695	1	/	/
*v*_12_	−0.193	−0.191	1	/
*G*_12_	0.647	0.972	−0.335	1

**Table 20:** Comparison of the probability distribution characteristics of the stiffness properties of H-GFRPs.

Stiffness	RTD			ETW		
	*μ*	*σ*	Distribution	*μ*	*σ*	Distribution
*E*_11_/GPa	56.45	3.89	Normal	3.99	0.069	Lognormal
*E*_22_/GPa	2.78	0.097	Lognormal	2.47	0.102	Lognormal
*v*_12_	0.289	0.011	Normal	0.289	0.011	Normal
*G*_12_/GPa	1.38	0.097	Lognormal	1.03	0.101	Lognormal

## Data Availability

The data presented in this study are available on request from the corresponding author.
